# A principled strategy for mapping enhancers to genes

**DOI:** 10.1038/s41598-019-47521-w

**Published:** 2019-07-30

**Authors:** Dongkyeong Kim, Hongjoo An, Randall S. Shearer, Mohamed Sharif, Chuandong Fan, Jin-ok Choi, Sun Ryu, Yungki Park

**Affiliations:** Hunter James Kelly Research Institute, Department of Biochemistry, Jacobs School of Medicine and Biomedical Sciences, State University of New York at Buffalo, Buffalo, NY 14203 USA

**Keywords:** Gene expression, Gene regulation, Epigenomics

## Abstract

Mapping enhancers to genes is a fundamental goal of modern biology. We have developed an innovative strategy that maps enhancers to genes in a principled manner. We illustrate its power by applying it to *Myrf*. Despite being a master regulator of oligodendrocytes, oligodendrocyte enhancers governing *Myrf* expression remain elusive. Since chromatin conformation capture studies have shown that a gene and its enhancer tend to be found in the same topologically associating domain (TAD), we started with the delineation of the *Myrf* TAD. A genome-wide map of putative oligodendrocyte enhancers uncovered 6 putative oligodendrocyte enhancers in the *Myrf* TAD, narrowing down the search space for *Myrf* enhancers from the entire genome to 6 loci in a principled manner. Epigenome editing experiments revealed that two of them govern *Myrf* expression for oligodendrocyte development. Our new method is simple, principled, and powerful, providing a systematic way to find enhancers that regulate the expression of a gene of interest. Since it can be applied to most cell types, it would greatly facilitate our effort to unravel transcriptional regulatory networks of diverse cell types.

## Introduction

Enhancers are short segments of DNA that orchestrate cell type-specific gene expression by serving as transcription factor binding platforms^1,2^. A fascinating yet perplexing feature of enhancers is that they are often far away from target genes. For example, the ZRS enhancer is almost 1 Mb away from its target gene *Shh*^3^. This has made it difficult to annotate enhancers to genes. For this reason, the traditional approach to finding enhancers for a gene is to find conserved sequence segments in its vicinity and to test whether they work as enhancers in cell culture and/or transgenic animals^4–9^. If they do, it is assumed that they would regulate the endogenous gene in the genomic context. Although it has been extensively used to successfully characterize putative enhancers for genes of interest, this traditional approach has a couple of shortcomings. First, one has to make an arbitrary decision about where and how far to look in the genome for conserved sequence segments. Is upstream 100 Kb enough? Or do we have to look both upstream and downstream for as far as 1 Mb? Second, the traditional approach just assumes a regulatory relationship between a gene and an enhancer based on a distance criterion. If they are close to each other, which is again an arbitrary decision, it assumes that the enhancer would regulate the gene.

To tackle this fundamental issue, we have developed a novel strategy that maps enhancers to genes in a principled manner. This paper illustrates its power by applying it to the gene *Myrf* encoding a master regulator of oligodendrocytes (OLs)^10–12^. A unique aspect of *Myrf*, compared to other crucial OL genes, is that it is highly expressed in differentiating OLs, but not in OL precursor cells (OPCs), indicating that *Myrf* expression marks the onset of OL differentiation. Consistently, gene expression analysis of multiple sclerosis lesions indicated that OLs stalled in their differentiation are those that fail to upregulate *Myrf* expression. Hence, elucidating how *Myrf* expression is activated in OLs holds a great promise for revealing the molecular events underlying OL differentiation and developing novel remyelination therapies. The identity of enhancers governing *Myrf* expression in OLs remains elusive, and this is why *Myrf* was chosen as the first target for our new method. The only information available about *Myrf* expression regulation is that there is an enhancer called ECR9 in the first intron of *Myrf*^5^, which was active in OLs and some other cell types when tested in transgenic mice. ECR9 has been assumed, but not proved, to regulate *Myrf* expression in OLs. Our new method shows that ECR9 and a novel OL enhancer jointly control *Myrf* expression for OL development, demonstrating its effectiveness. Importantly, our new strategy can be applied to other genes and cell types and is expected to greatly accelerate our effort to unravel transcriptional regulatory networks of diverse cell types.

## Results

### Overview: A principled strategy for mapping enhancers to genes

**Figure 1:**
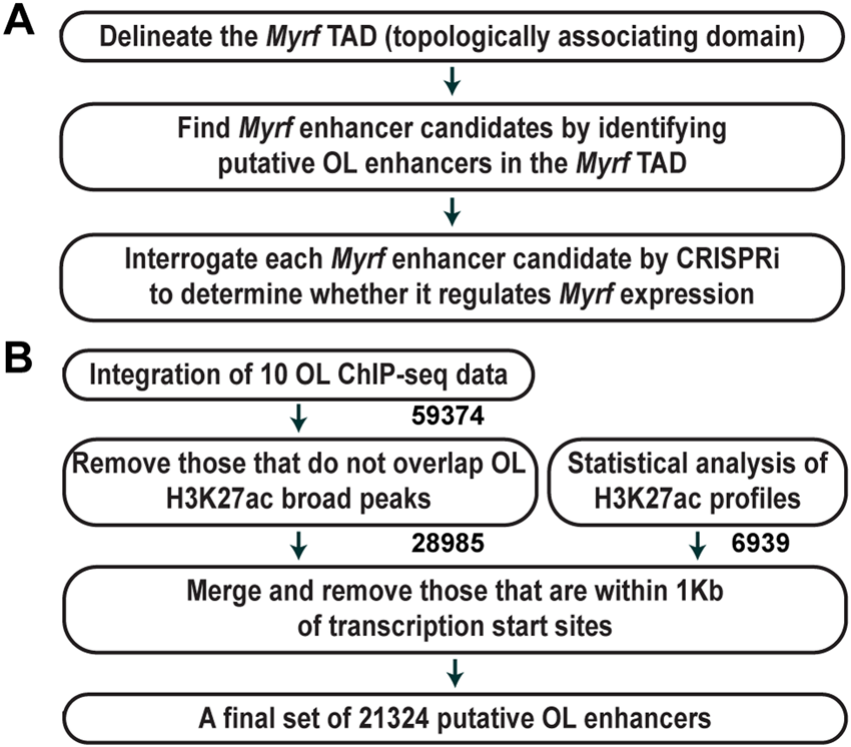
Schemes for identifying *Myrf* enhancers and deriving a genome-wide map of putative OL enhancers. (**A**) A principled approach that we propose to find *Myrf* enhancers. (**B**) An overview of computational analysis that generates a genome-wide map of 21324 putative promoter-distal OL enhancers.

Our strategy consists of three steps (Fig. 1A), and we will illustrate them by using *Myrf* as an exemplary case. First, a paradigm-shift discovery from chromatin conformation capture studies is that a gene and its enhancer tend to be found in the same topologically associating domain (TAD), a fundamental unit of genome organization and function^13–17^. With the TAD knowledge, one does not have to make an arbitrary decision about where and how far to look in the genome for enhancer candidates. The TAD information allows one to narrow down the enhancer search space in a principled manner. There are two features of a TAD that need to be distinguished – internal detail and boundaries^18^. The internal detail of a TAD reflects cell type-specific interactions among genes and enhancers, and it differs between cell types. In contrast, the boundaries of a TAD tend to be conserved between cell types and species^13^. Thus, even if there is no chromatin interaction data for OLs, we can still delineate the *Myrf* TAD by analyzing public chromatin interaction data for other cell types, as shown below. Second, we identify putative OL enhancers in the *Myrf* TAD, which are *Myrf* enhancer candidates because they are in the same TAD as *Myrf*. By comparing the *Myrf* TAD with a genome-wide map of putative OL enhancers (see below), we were able to identify 6 *Myrf* enhancer candidates. Third, we interrogate the *Myrf* enhancer candidates with CRISPRi^19–22^, a cutting-edge epigenome editing technique, to determine whether they govern *Myrf* expression in OLs. A definitive way of proving an enhancer-target gene relationship is to demonstrate that the inactivation of the enhancer downregulates the target gene. Unfortunately, this seemingly simple experiment used to be almost impossible due to the lack of tools that manipulate enhancer activity in the genomic context. This is why the traditional approach just assumes a regulatory relationship between a gene and an enhancer based on a distance criterion. With the advent of CRISPRi, we can inactivate enhancers in the genomic context, linking enhancers to target genes on the basis of experimental proof of causality in gene expression. Below, we describe each step in detail and demonstrate how principled and powerful this new strategy is for finding enhancers that govern the expression of a given gene.

### TAD analysis for *Myrf*

**Figure 2:**
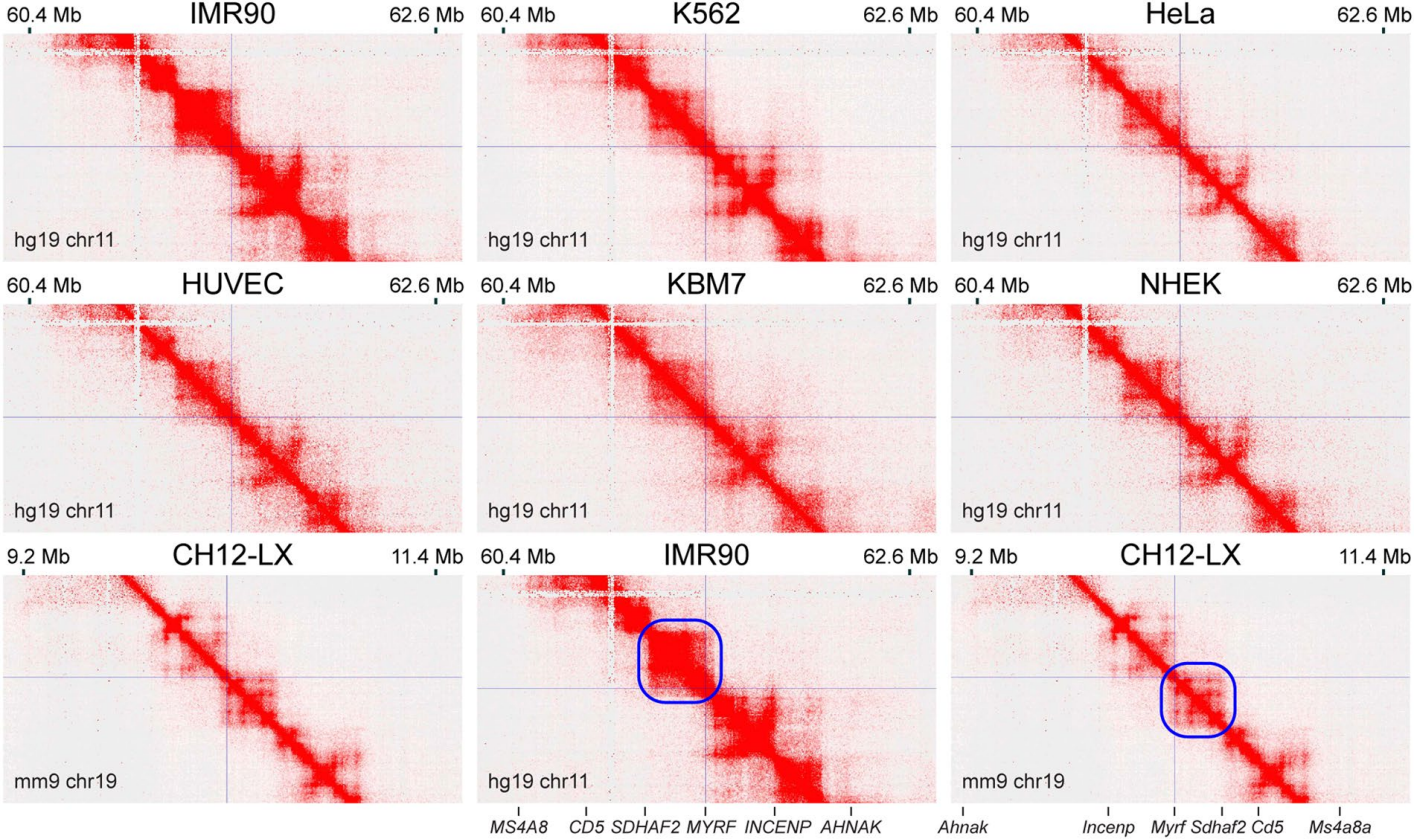
The *Myrf* TAD is conserved between cell types and species. The publicly available 5 Kb-resolution Hi-C data for 7 diverse cell types from human and mouse^23^. The interaction frequency between two loci is indicated by color: white means no interaction, and red the strongest possible interaction. The *Myrf* promoter position is marked by thin crossing lines. The *Myrf* TAD is marked by a blue box for CH12-LX. The corresponding TAD for human is marked for the IMR90 data. IMR90: lung fibroblast. K562 and KBM7: chronic myelogenous leukemia cells. HeLa: cervical cancer cell. HUVEC: human umbilical vein endothelial cell. NHEK: normal human epidermal keratinocyte. CH12-LX: murine CH12 B cell lymphoma cell. This figure was generated by Juicebox^52,53^.

In order to delineate the *Myrf* TAD, we analyzed publicly available kilobase-resolution Hi-C data for 7 diverse cell types from human and mouse^23^. The location of the *MYRF/Myrf* promoter is indicated by thin crossing lines in Fig. 2. The TAD organization around *MYRF* is well defined and conserved between different cell types (Fig. 2). The *MYRF* syntenic region is flipped in mouse compared to human. Strikingly, this flip is also mirrored in the *Myrf* TAD (CH12-LX, Fig. 2), highlighting a high degree conservation of the *Myrf* TAD through evolution. The *Myrf* TAD is about 300 Kb long (a thick blue square in CH12-LX), suggesting that critical *Myrf* enhancers would be found in the region spanning downstream 30 Kb and upstream 270 Kb of *Myrf*.

### A genome-wide map of 21324 putative promoter-distal OL enhancers

Although the TAD analysis helps to narrow down the search space for *Myrf* enhancers, the *Myrf* TAD is still quite large. It is impractical to apply CRISPRi (CRISPR interference) to such a large genomic region for enhancer discovery (see below). To remedy this situation, we have derived a genome-wide map of putative OL enhancers by analyzing public OL ChIP-seq (chromatin immunoprecipitation coupled with high-throughput sequencing) data, with the idea being that the genome-wide map of putative OL enhancers would reveal putative OL enhancers in the *Myrf* TAD, which are *Myrf* enhancer candidates because they are in the same TAD as *Myrf*.

**Figure 3:**
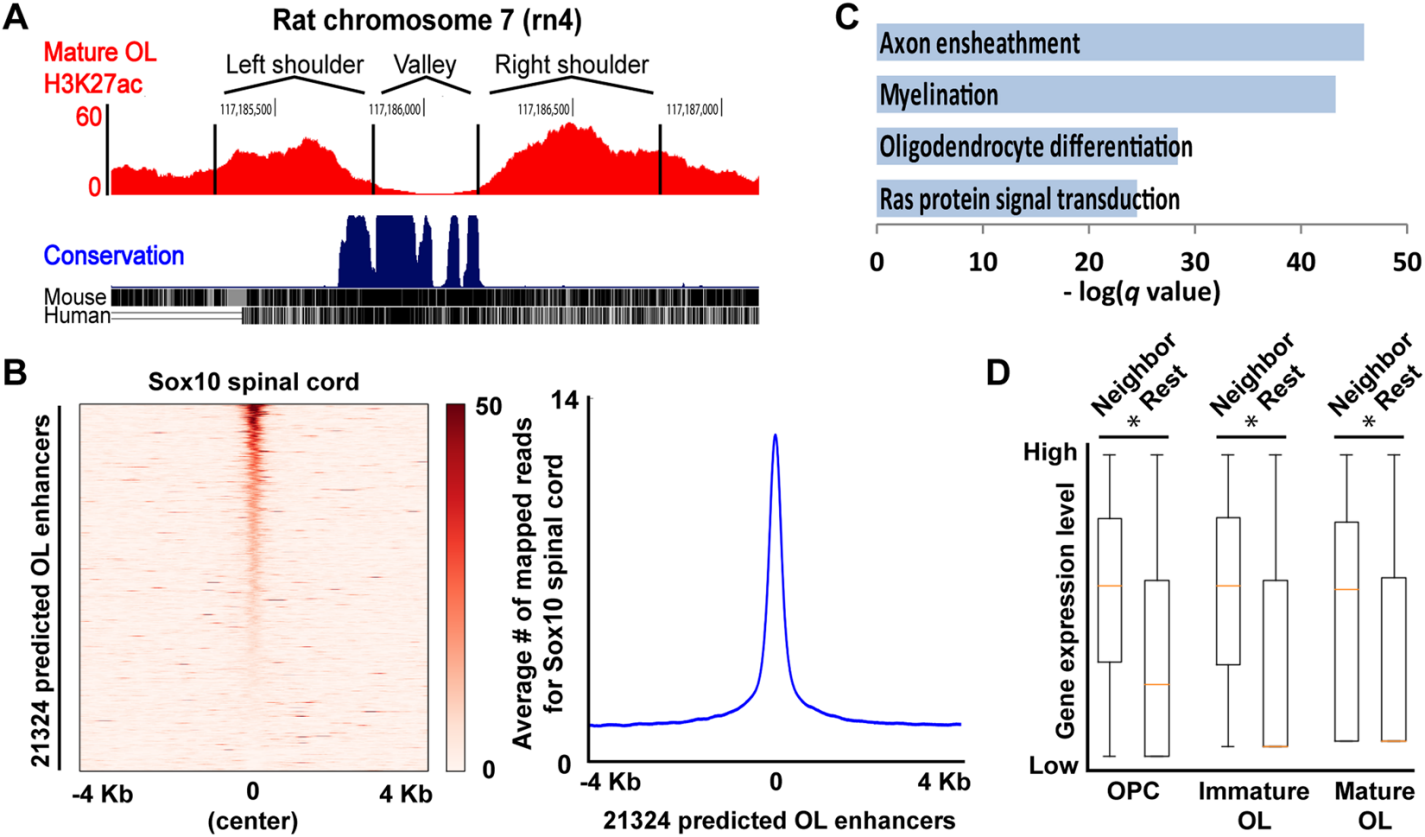
Characterization of the genome-wide map of putative OL enhancers. (**A**) A typical peak-valley-peak pattern for an enhancer in H3K27ac ChIP-seq data. (**B**) The Sox10 spinal cord ChIP-seq data^30^ were aligned with the 21324 putative OL enhancers. (**C**) Gene ontology analysis by GREAT^33^. (**D**) The brain RNA-seq database^34^ was looked up to estimate the expression level of genes in *in vivo* OL lineage cells. A box plot shows that genes neighboring the putative OL enhancers, as defined by GREAT, are expressed at a significantly higher level than the rest. *p value ≈ 0 by the Mann–Whitney–Wilcoxon test corrected by the Bonferroni procedure.

To predict OL enhancers on a genome-wide scale, we first identified 59374 genomic regions bound by Olig2, Sox10, Myrf, Brg1, Tcf7l2, or Chd7 (Fig. 1B)^24–27^. Of note, all these ChIP-seq data are from cultured rat OL lineage cells. Second, they were filtered by the H3K27ac broad peaks of rat OL lineage cells^24^ because H3K27ac marks active enhancers^28^, leaving 28985 genomic regions. Third, since ChIP-seq data are not available for all OL transcription factors, we have developed a statistical method that predicts enhancers independent of transcription factor ChIP-seq data. We took advantage of a unique feature of enhancers in H3K27ac ChIP-seq profiles. Since enhancers are bound by transcription factors, they are usually depleted of nucleosomes. Hence, although H3K27ac is known to mark active enhancers, its enrichment is found in shoulders flanking an enhancer rather than in the enhancer itself. This is why a peak-valley-peak pattern is observed for enhancers in H3K27ac ChIP-seq data (Fig. 3A). However, a reasonable null model would posit that H3K27ac ChIP-seq reads are distributed randomly (*i.e*., uniformly). Under the null model, the peak-valley-peak pattern of H3K27ac ChIP-seq reads for an enhancer would be unlikely, and this deviation from the null model can be quantified by the binomial cumulative distribution function (see Methods). Analysis of the public H3K27ac ChIP-seq data for OL lineage cells^24^ with the binomial cumulative distribution function revealed that 5804 of the 45212 H3K27ac broad peaks have at least one incidence of the peak-valley-peak pattern, leading to the prediction of 6939 OL enhancers. Interestingly, the 5804 broad peaks display a significantly higher level of H3K27ac signals than the rest (Fig. S1A). Finally, the 28985 genomic regions from the second step were merged with the 6939 putative OL enhancers from the third step (Fig. 1B). From the resulting set, we removed those within 1 Kb of transcription start sites, ending up with a final set of 21324 putative promoter-distal OL enhancers.

We examined several aspects of the predicted OL enhancers to estimate its quality. First, we examined the size distribution of putative OL enhancers with the H3K27ac peak-valley-peak configuration. 3612 of the 21324 putative OL enhancers are overlaid with the H3K27ac peak-valley-peak pattern (Fig. S1B). The average size of the 3612 putative OL enhancers, as defined by the distance between the half-points of the flanking H3K27ac peaks, is about 390 base pairs (Fig. S1B). In other words, the 3612 putative OL enhancers are characterized by a 390 base pair-long nucleosome-depleted region, which is in line with a previous estimate^29^. Nucleosome depletion for the 3612 putative OL enhancers is likely due to the competitive DNA binding of transcription factors because transcription factor ChIP-seq peaks fall within the 390 base pair-long region (Fig. S1C,D). Second, we looked into the *in vivo* relevance of the 21324 putative OL enhancers. The Svaren laboratory published Sox10 ChIP-seq data for the spinal cord^30^. Our putative OL enhancers were derived independent of it. Sox10 is a key transcriptional regulator of OL lineage cells^5,31,32^, and a subset of important OL enhancers is expected to be marked by it. For Sox10 ChIP-seq peaks that are within ±4 Kb of the putative OL enhancers, about 85% of their ChIP-seq reads are mapped to the putative OL enhancers (defined as 390 base pairs long, Figs 3B and S1E). This high concentration of Sox10 ChIP-seq reads in the putative OL enhancers is not expected by chance (*p* value ≈ 0 by the binomial cumulative distribution function), supporting the *in vivo* relevance of the putative OL enhancers. Third, gene ontology (GO) analysis by GREAT^33^ indicates that the putative OL enhancers are significantly associated with GO terms related to OL development and central nervous system (CNS) myelination (Fig. 3C). Fourth, genes neighboring the putative OL enhancers, as defined by GREAT, tend to be expressed at a higher level in *in vivo* OL lineage cells than the rest^34^ (Fig. 3D), suggesting that our putative OL enhancers are active in the mouse brain. Overall, these results suggest a high quality for our putative OL enhancers.

### Identification and CRISPRi analysis of 6 *Myrf* enhancer candidates

**Figure 4:**
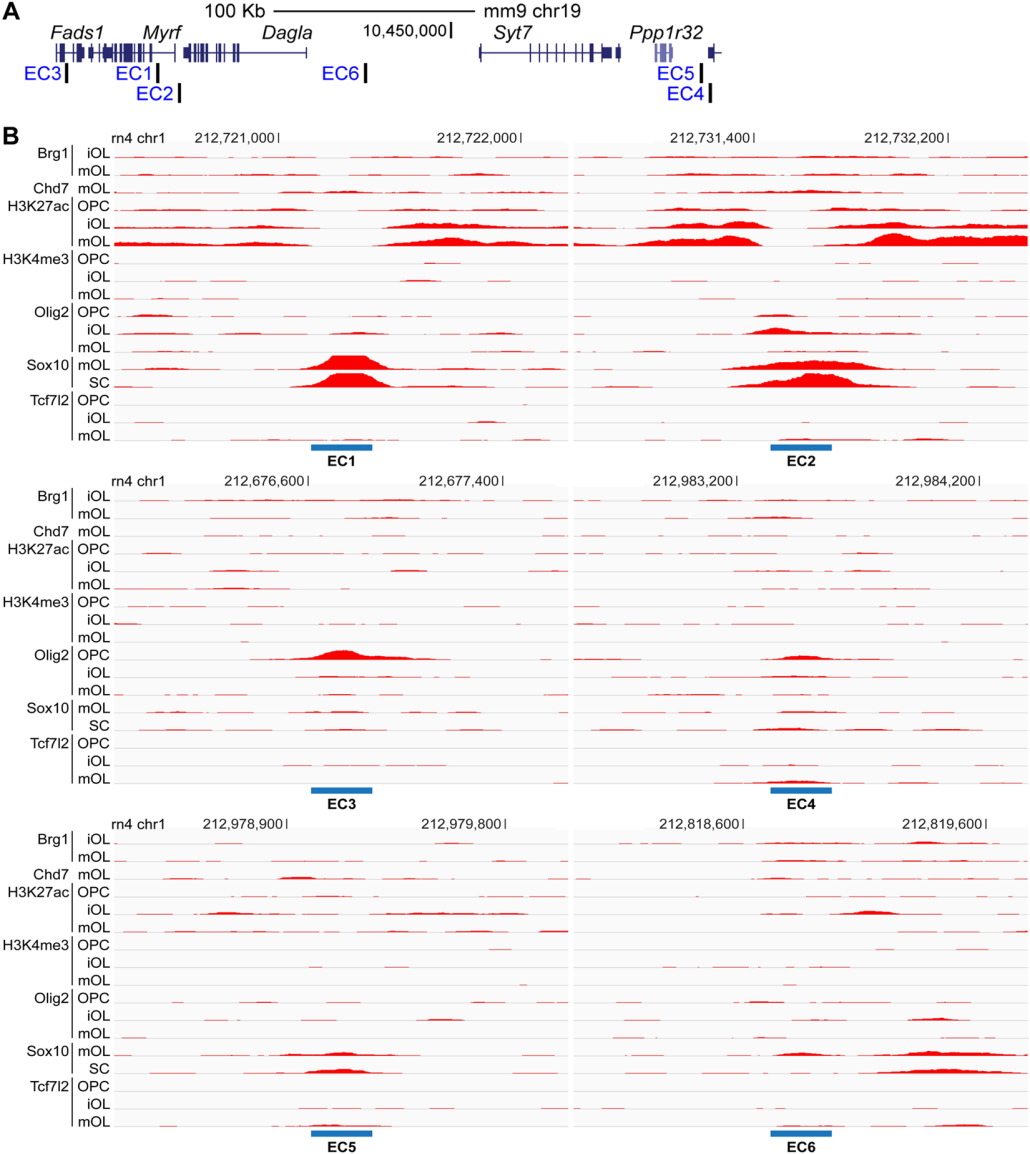
Six *Myrf* enhancer candidates. (**A**) Genomic locations of the 6 *Myrf* enhancer candidates. (**B**) Rat OL ChIP-seq data underlying the 6 *Myrf* enhancer candidates. iOL: immature OL. mOL: mature OL. OPC: oligodendrocyte precursor cells. SC: spinal cord.

We compared our genome-wide map of putative OL enhancers with the *Myrf* TAD, finding 6 putative OL enhancers in the *Myrf* TAD. Since these 6 putative OL enhancers are in the same TAD as *Myrf*, they are *Myrf* enhancer candidates (EC1, 2, 3, 4, 5, and 6 in Fig. 4A). The 6 putative OL enhancers in the *Myrf* TAD were sorted by the strength of the underlying ChIP-seq data (Fig. 4B) and named accordingly. The one with the strongest evidence was named EC1, and the one with the weakest evidence EC6. The two best Myrf enhancer candidates (EC1 and EC2) are overlaid with the H3K27ac peak-valley-peak pattern. In addition, EC1 and EC2 are strongly bound by Sox10. The other four enhancer candidates are only associated with weak binding of Olig2 and Sox10. Four *Myrf* enhancer candidates are in the upstream region (EC2, EC4, EC5, and EC6), one in the first intron (EC1), and one in the downstream region (EC3). The one in the first intron (EC1 in Fig. 4A) is the same as ECR9 discovered by the Wegner laboratory on the basis of interspecies sequence conservation^5^. Bound by Sox10, ECR9 was shown to work as an enhancer in OL lineage cells of transgenic mice. Thus, ECR9 has been assumed, but not proved, to regulate *Myrf* expression in OLs. Our approach to identifying *Myrf* enhancer candidates does not rely on sequence conservation (Fig. 1B). Nonetheless, it successfully recovered ECR9, a known enhancer in the vicinity of *Myrf*, attesting to its good sensitivity.

When deriving the genome-wide map of putative OL enhancers, we used lenient criteria to minimize false negatives. Consequently, the six *Myrf* enhancer candidates may not all be OL enhancers. Even if they are OL enhancers, they may not regulate *Myrf* expression. Whether they are OL enhancers that govern *Myrf* expression needs to be determined experimentally. To this end, we resorted to CRISPRi in which dCas9-KRAB, a fusion protein between a nuclease-null Cas9 (dCas9) and a KRAB (Krüppel associated box) domain, is targeted to a specific locus by single guide RNAs (sgRNAs)^19–22^. When targeted to a promoter, dCas9-KRAB silences it, decreasing gene expression. When targeted to an enhancer, dCas9-KRAB inactivates it, which in turn downregulates target genes. To avoid the danger of false positive and false negative, each *Myrf* enhancer candidate was tested with at least 5 independent sgRNAs. In our CRISPRi experiment, dCas9-KRAB and sgRNA plasmids were transfected into primary mouse OPCs purified by immunopanning^35,36^, and transfected OPCs were cultured in a differentiation condition for 3 days to induce their differentiation into OLs and *Myrf* expression^35^. To easily monitor the expression level of *Myrf*, Rffl (an OL enhancer in the *Rffl* locus [rn4 chr10:71034166–71034749], which is a well-characterized Myrf luciferase reporter^25,37,38^) was co-transfected. Since co-transfection efficiency is high and luciferase assay extraordinarily sensitive, co-transfection of Rffl allowed us to detect changes in *Myrf* expression in the few transfected cells by a simple luciferase assay without selecting them. Of note, it remains unknown whether Rffl governs the expression of *Rffl*, which does not concern the current study.

**Figure 5:**
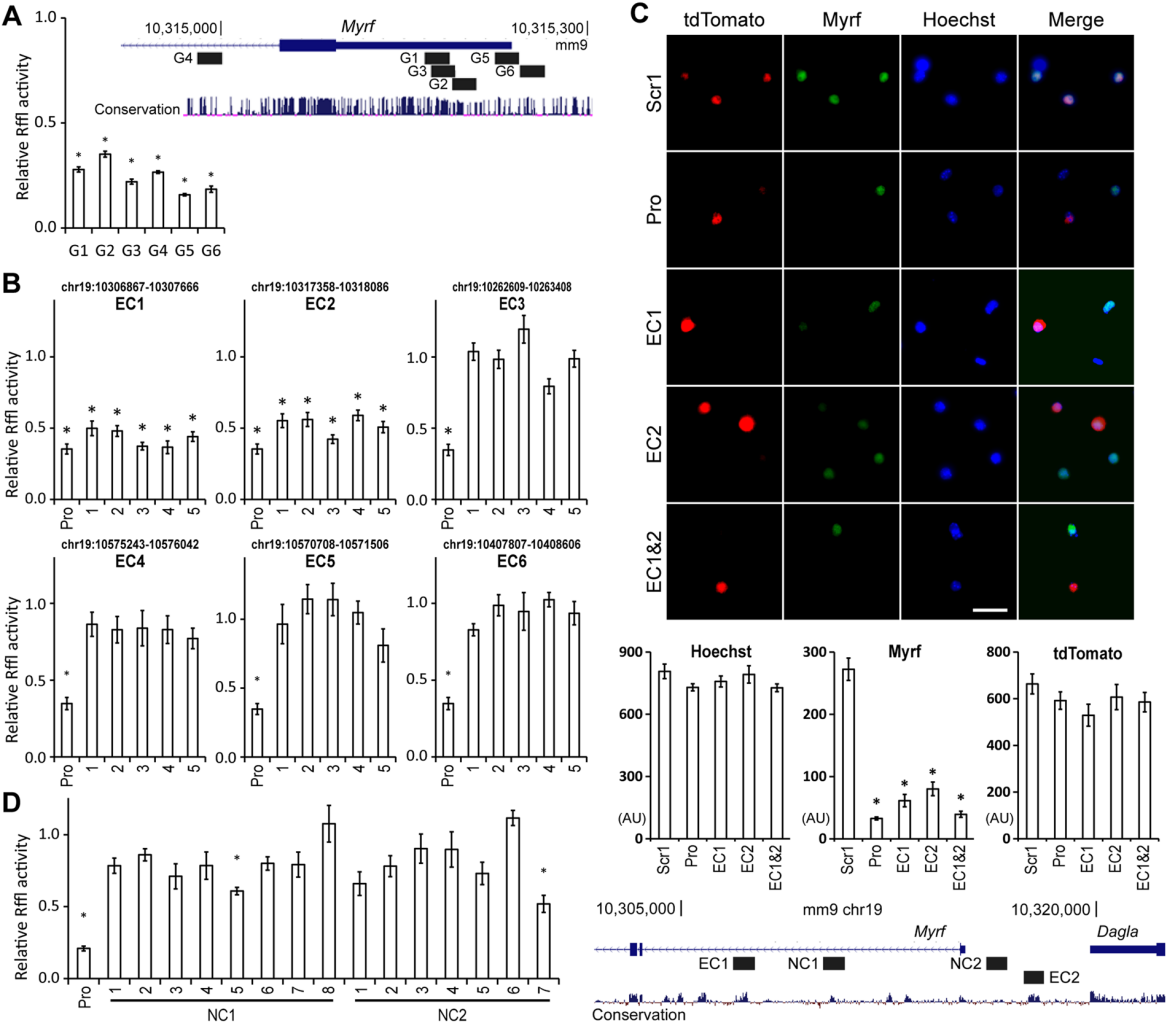
Interrogation of the 6 *Myrf* enhancer candidates by CRISPRi. (**A**) CRISPRi validation for the *Myrf* locus. The expression level of *Myrf* was estimated by the reporter activity of Rffl, a highly specific and sensitive Myrf luciferase reporter^25,37^. The Rffl activity for each sgRNA was divided by the average Rffl activity for Scr1 and Scr2 to get the relative Rffl activity. For each sgRNA, the mean and standard error are shown. *p value < 1.4 × 10^−4^ by two-sided one sample Student’s *t* test corrected by the Bonferroni procedure (n = 4). (**B**) For each of the 6 *Myrf* enhancer candidates, 5 independent sgRNAs were used. For each sgRNA, the mean and standard error of the relative Rffl activity are shown. *p value < 3.3 × 10^−2^ by two-sided one sample Student’s *t* test corrected by the Bonferroni procedure (n = 9). (**C**) The signal from each fluorescence channel was quantified for individual cells by CellProfiler^40^. The number of cells analyzed is as follows: Scr1 (91), Pro (79), EC1 (42), EC2 (46), EC1&2 (82). Scale bar, 20 µm. AU: arbitrary unit. Targeting dCas9-KRAB to the *Myrf* promoter (Pro, by G5 in panel A), EC1 (by 5 in panel B), EC2 (by 4 in panel B), or EC1&2 (by 5 and 4 in panel B, respectively) led to a significant drop in *Myrf* expression. *p value < 4.6 × 10^−10^ by two-sided unpaired Student’s *t* test corrected by the Bonferroni procedure (comparison with Scr1). (**D**) Epigenome editing analysis was repeated for two negative control regions, NC1 and NC2. The mean and standard error for 8 and 7 sgRNAs that tile NC1 and NC2, respectively, are shown. *p value < 2.4 × 10^−3^ by two-sided one sample Student’s *t* test corrected by the Bonferroni procedure (n = 8).

Before interrogating the six *Myrf* enhancer candidates with CRISPRi, we validated CRISPRi for the *Myrf* locus. We designed six sgRNAs for the *Myrf* promoter and tested them in Oli-neu cells, a widely used OL cell line^39^ (G1-G6 in Fig. 5A). Scr1 and Scr2 are non-targeting negative control sgRNAs. When dCas9-KRAB was targeted by Scr2, there was no change in *Myrf* expression compared to Scr1, as expected (Fig. S2). Since Scr1 and Scr2 allow us to estimate non-specific effects associated with the expression of the CRISPRi components, we normalized all our data to the average of Scr1 and Scr2 for robust statistical analysis. When dCas9-KRAB was delivered to the *Myrf* promoter by the 6 sgRNAs, *Myrf* expression went down by 65–85% compared to the average of Scr1 and Scr2 (Fig. 5A), demonstrating that dCas9-KRAB works well for *Myrf*. Of the 6 sgRNAs, G5 was the most potent, and we used it throughout our study as a positive control. Having validated CRISPRi for *Myrf*, we tiled each *Myrf* enhancer candidate with 5 independent sgRNAs and used them to determine whether it regulates *Myrf* expression in primary mouse OLs. For a true *Myrf* enhancer, most of the 5 sgRNAs would lead to a significant drop in *Myrf* expression. For other genomic regions, few of the 5 sgRNAs would result in such change. Targeting dCas9-KRAB to EC1 or EC2 by the 5 sgRNAs significantly downregulated *Myrf* expression (Fig. 5B). In contrast, delivery of dCas9-KRAB to the other four *Myrf* enhancer candidates did not affect *Myrf* expression. Thus, the luciferase assay-based epigenome editing analysis indicates that EC1 and EC2 govern *Myrf* expression in primary mouse OLs. We also got the same results with Oli-neu cells (Fig. S2).

To corroborate the above result, we performed a quantitative immunofluorescence experiment. A plasmid expressing dCas9-KRAB and tdTomato was transfected into primary mouse OPCs, together with sgRNA plasmids. Transfected OPCs were cultured in the differentiation condition for 3 days to induce their differentiation into OLs. They were then stained for Myrf and tdTomato (identifying transfected cells). The Myrf antibody used for this experiment has been previously validated for immunofluorescence^5,12^. We further confirmed it by comparing its immunofluorescence with that of a well-established Flag antibody for Flag-tagged Myrf constructs in Oli-neu cells (Fig. S3). Co-expression of dCas9-KRAB with Scr1 did not interfere with *Myrf* expression (Fig. 5C). However, when dCas9-KRAB was targeted to the *Myrf* promoter (Pro, Fig. 5C), *Myrf* expression was significantly downregulated. For an objective image analysis, the signal from each fluorescence channel (Hoechst, Myrf, and tdTomato) was quantified for individual OLs by CellProfiler^40^. This revealed that Hoechst and tdTomato signals were comparable across the samples. In contrast, Myrf signals were much lower when dCas9-KRAB was targeted to the *Myrf* promoter, EC1, EC2, or EC1&2 (Fig. 5C). These observations reinforce our epigenome editing analysis that EC1 and EC2 are required for the expression of *Myrf*.

### Confirming the epigenome editing analysis

EC1 and EC2 happen to be the closest ones to *Myrf*. EC1 is in the first intron of *Myrf*, and EC2 is located 2 Kb upstream of *Myrf*. Creating repressive chromatin in an intron may hinder transcriptional elongation. Similarly, repressive epigenetic modifications induced by dCas9-KRAB for EC2 may spread to the *Myrf* promoter. Hence, it is possible that EC1 and EC2 came out positive in our epigenome editing analysis due to the non-specific effect of dCas9-KRAB. An exhaustive analysis for the *GATA1* and *MYC* loci by dCas9-KRAB has shown that dCas9-KRAB is highly specific^21^, not displaying non-specific effects in promoter upstream regions and gene bodies (Fig. S4). This known specificity of dCas9-KRAB-mediated epigenome editing makes it unlikely that EC1 and EC2 are false positives. To experimentally confirm this conjecture, we repeated epigenome editing analysis for two negative control regions, NC1 and NC2 (Fig. 5D). NC1 is located between EC1 and the *Myrf* promoter. NC2 is found between EC2 and *Myrf*. No significant peak is observed for them in the rat OL ChIP-seq data (data not shown) and the public single-cell ATAC-seq data (see below). These features make NC1 and NC2 suitable negative controls. Of the 8 sgRNAs tested for NC1, only one came out positive (Fig. 5D). For NC2, only one came out positive from the 7 tested sgRNAs. These results are consistent with the known specificity of dCas9-KRAB and indicate that the positive epigenome editing results for EC1 and EC2 cannot be explained by non-specific effects of dCas9-KRAB.

**Figure 6:**
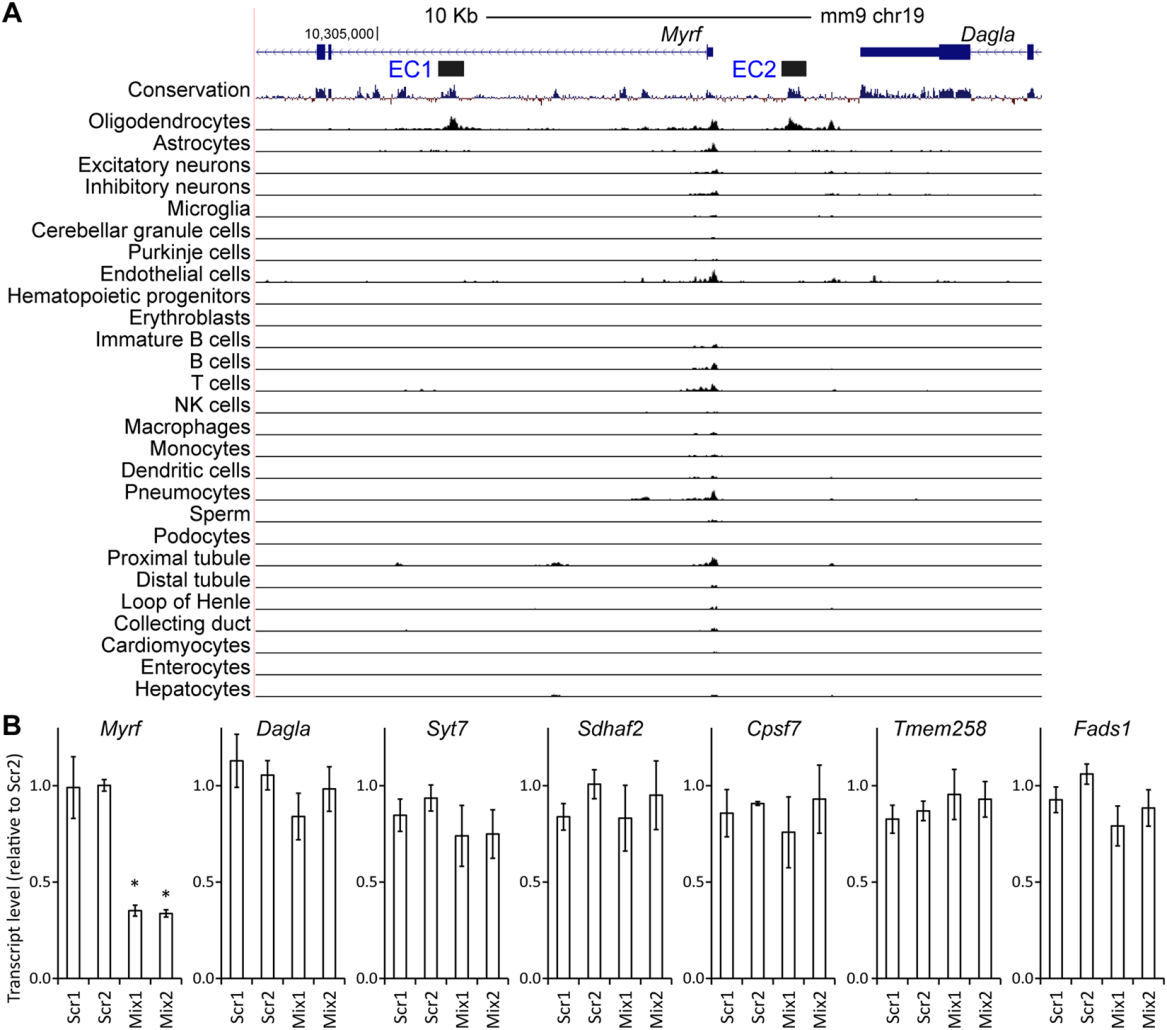
Specificity of EC1 and EC2. (**A**) Single-cell ATAC-seq data for 13 mouse tissues^41^, which were clustered into 27 broadly defined cell types. (**B**) RT-qPCR data showing that CRISPRi silencing of EC1 and EC2 downregulates *Myrf* expression but does not impact the expression of nearby genes. Shown are the mean and standard error after normalization by Scr2. *p value < 1.3 × 10^−5^ by two-sided unpaired Student’s *t* test corrected by the Bonferroni procedure (n = 4).

To gain further support for this conclusion, we analyzed public single-cell ATAC-seq data. By using a single-cell ATAC-seq method, Shendure and co-workers determined chromatin accessibility for 13 different mouse tissues at a single cell resolution^41^. The resulting data were clustered into 27 broadly defined cell types. Notably, of the first intron of *Myrf*, EC1 is uniquely accessible (Fig. 6A). Likewise, EC2 is a specific peak in the upstream region of *Myrf*. These observations underscore the remarkable specificity of EC1 and EC2 despite their proximity to the *Myrf* promoter. We also analyzed the single-cell ATAC-seq data for the other four negative *Myrf* enhancer candidates (EC3, EC4, EC5, and EC6). In agreement with our epigenome editing analysis, they were not accessible in OLs (Fig. S5). Overall, these analyses reinforce our epigenome editing analysis that EC1 and EC2 are the only positive ones from the 6 *Myrf* enhancer candidates.

Having confirmed that EC1 and EC2 govern *Myrf* expression, we wondered whether EC1 and EC2 are specific to *Myrf*. In other words, do EC1 and EC2 also regulate the expression of other genes in the vicinity of *Myrf*? To address this issue, we generated four Oli-neu cell lines where both EC1 and EC2 can be inducibly silenced by CRISPRi. The first and second cell lines express Scr1 and Scr2, respectively. The third cell line expresses two sgRNAs that deliver dCas9-KRAB to EC1 and EC2 (4 of Fig. 5B for EC1 and 3 of Fig. 5B for EC2, called Mix1 in Fig. 6B). To corroborate the results for this cell line, the fourth cell line expresses two other sgRNAs that deliver dCas9-KRAB to EC1 and EC2 (3 of Fig. 5B for EC1 and 5 of Fig. 5B for EC2, called Mix2 in Fig. 6B). These four Oli-neu cell lines express dCas9-KRAB in a doxycycline-dependent manner. To execute CRISPRi, doxycycline was added to the culture media for 2 days before RNA harvest. The expression level of *Myrf* and nearby genes that are expressed in Oli-neu cells was quantified by RT-qPCR where *Gapdh* was used as a control. When dCas9-KRAB was targeted to EC1 and EC2 by Mix1 or Mix2, *Myrf* expression went down by more than 65% (*p < 1.3 × 10^−5^ by unpaired two-sided Student’s t test corrected by the Bonferroni procedure, Fig. 6B), consistent with the above epigenome editing analyses. We analyzed the same RNA samples for other genes, finding no significant change in their expression levels (Fig. 6B). Collectively, these results demonstrate that EC1 and EC2 are highly specific to *Myrf*.

### EC1 and EC2 are OL-specific enhancers

**Figure 7:**
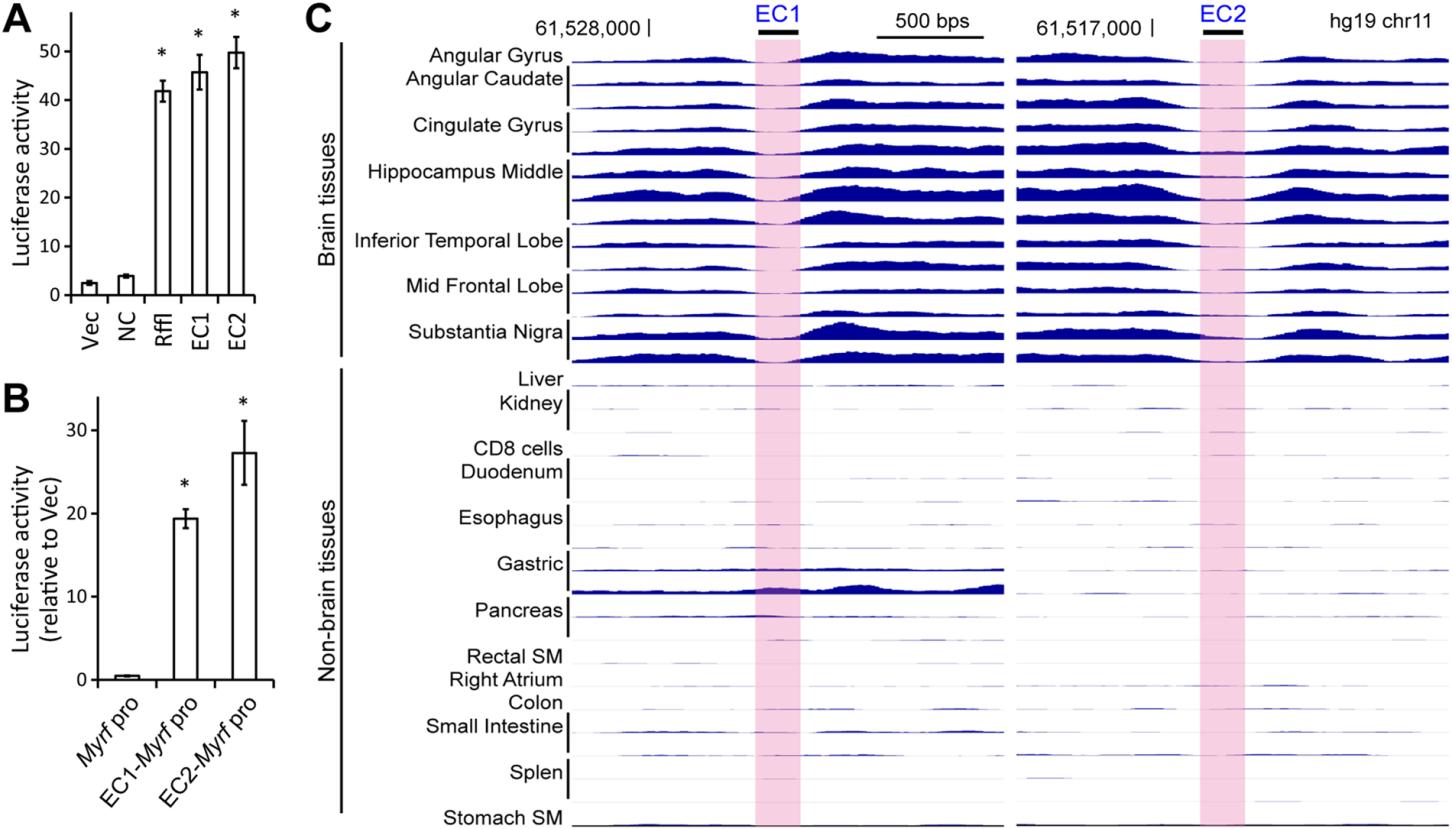
EC1 and EC2 are OL-specific enhancers. (**A**) EC1 and EC2 are as good at activating the SV40 promoter of pGL3-promoter as Rffl, a known OL enhancer. The mean and standard error of the luciferase activity are shown for each construct. *p value < 4.3 × 10^−8^ by two-sided unpaired Student’s *t* test corrected by the Bonferroni procedure (comparison with Vec, n = 8). (**B**) EC1 and EC2 activate the *Myrf* promoter. The SV40 promoter of pGL3-promoter was replaced by the *Myrf* promoter, and the luciferase assay repeated. Shown are the mean and standard error relative to Vec (pGL3-promoter). *p value < 3.4 × 10^−3^ by two-sided unpaired Student’s *t* test corrected by the Bonferroni procedure (comparison with the Myrf promoter alone “*Myrf* pro”, n = 4). (**C**) The H3K27ac ChIP-seq data for EC1 and EC2 from the NIH Roadmap Epigenomics Project^42^. A complete dataset for EC1 and EC2 that encompasses more diverse tissues and cell types is available in Fig. S6. SM: smooth muscle.

EC1, which is the same as ECR9, was previously shown to be activated by Sox10 and work as an enhancer in OLs^5^. Thus, there is strong evidence supporting its enhancer identity, although it remains unknown whether it is an OL-specific enhancer and whether it is also active in the human CNS. Regarding EC2, virtually nothing is known. In order to address these issues, we first tested the enhancer activity of EC1 and EC2 in primary mouse OLs. EC1 and EC2 were cloned into pGL3-promoter, and they were transfected into primary mouse OPCs. Transfected OPCs were cultured in the differentiation condition for 3 days to induce their differentiation. The SV40 promoter of pGL3-promoter had a very low basal activity in differentiating OLs (Vec in Fig. 7A). As a control, a genomic segment around *Myrf* that is not thought to work as an OL enhancer based on the OL ChIP-seq data (mm9 chr19:10318801–10319600) was cloned into pGL3-promoter (NC in Fig. 7A). NC failed to activate the SV40 promoter. In contrast, Rffl significantly activated the SV40 promoter. EC1 and EC2 were as powerful as Rffl (Fig. 7A), indicating that they work as enhancers in OLs. The same results were obtained when we replaced the SV40 promoter in pGL3-promoter with a minimal *Myrf* promoter (mm9 chr19:10315178–10315547)^5^. The minimal *Myrf* promoter had a very low basal activity in OLs (*Myrf* pro in Fig. 7B). However, its activity was significantly upregulated when EC1 or EC2 was placed upstream of it (EC1-*Myrf* pro and EC2-*Myrf* pro in Fig. 7B).

Having confirmed the OL enhancer activity of EC1 and EC2, we analyzed the single-cell ATAC-seq data to determine whether they are OL-specific enhancers. Of the 27 broadly defined cell types, EC1 and EC2 are accessible only in OLs (Fig. 6A), indicating that they work as OL-specific enhancers in mouse. We also looked up the H3K27ac ChIP-seq data from the NIH Roadmap Epigenomics Project^42^ to determine the tissue specificity and conservation of EC1 and EC2 in human. Consistent with the mouse single-cell ATAC-seq data, EC1 and EC2 exhibit the peak-valley-peak pattern only in the brain tissue (Fig. 7C; see also Fig. S6). EC1 and EC2 are also overlaid with the H3K4me1 peak-valley-peak pattern (Fig. S7; H3K4me1 marking enhancers^28^). EC1 and EC2 are not marked by H3K9me3 and H3K27me3 in the human brain (Figs S8,S9; both being repressive histone marks). Taken together, we conclude that EC1 and EC2 are OL-specific enhancers that are conserved between human and mouse.

### EC1 and EC2 are required for OL differentiation

**Figure 8:**
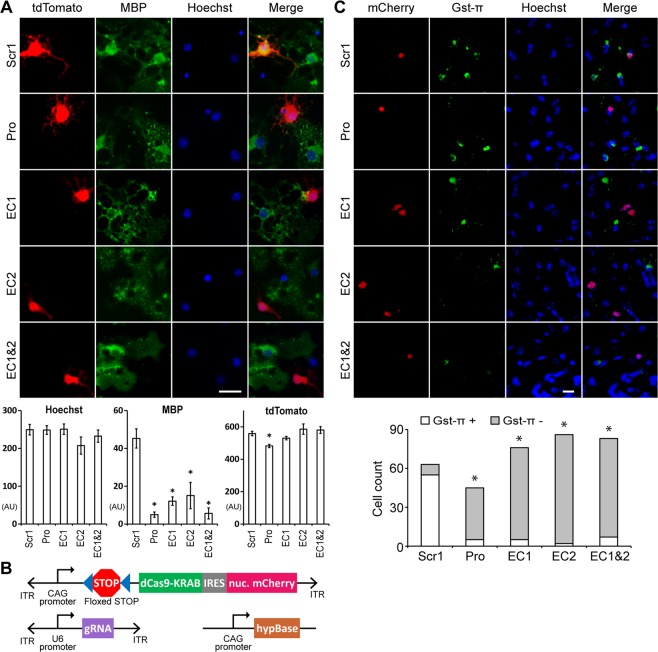
EC1 and EC2 are required for OL differentiation. (**A**) Targeting dCas9-KRAB to the *Myrf* promoter (Pro, by G5 in Fig. 5A), EC1 (by 5 in Fig. 5B), EC2 (by 4 in Fig. 5B), or EC1&2 (by 5 and 4 in Fig. 5B, respectively) decreased *Mbp* expression in primary mouse OLs. Scale bar, 20 µm. The signal from each fluorescence channel was quantified for individual cells by CellProfiler. AU: arbitrary unit. *p value < 1.6 × 10^−2^ by two-sided unpaired Student’s *t* test corrected by the Bonferroni procedure (comparison with Scr1). The number of cells analyzed is as follows: Scr1 (165), Pro (142), EC1 (177), EC2 (46), and EC1&2 (99). (**B**) Three plasmids electroporated into SVZ neural stem cells of P1 *Cnp*^*Cre/+*^ mice. ITR: piggyBac inverted terminal repeat. hypBase: hyperactive piggyBac transposase^45^. IRES: internal ribosome entry site. (**C**) Targeting dCas9-KRAB to the *Myrf* promoter (Pro, by G5 in Fig. 5A), EC1 (by 5 in Fig. 5B), EC2 (by 4 in Fig. 5B), or EC1&2 (by 5 and 4 in Fig. 5B, respectively) downregulated the expression of *Gst-π* in OL lineage cells in the mouse brain. Scale bar, 20 µm. *p value < 3.6 × 10^−30^ by the cumulative binomial distribution function corrected by the Bonferroni procedure. See Fig. S11B for the uncropped brain section images.

Since *Myrf* is indispensable for OL development^10^ and EC1 and EC2 are essential for the expression of *Myrf* in OLs, we hypothesized that EC1 and EC2 would be required for OL differentiation. To test this hypothesis, primary mouse OPCs were transfected with two plasmids: one co-expressing dCas9-KRAB and tdTomato, and the other expressing sgRNAs. Transfected OPCs were cultured in the differentiation condition for 3 days and stained for tdTomato and myelin basic protein (MBP, a mature OL marker). The signal from each fluorescence channel was quantified for individual cells by CellProfiler. The quantitative image analysis revealed that targeting dCas9-KRAB to the *Myrf* promoter, EC1, EC2, or EC1&2 significantly decreases *Mbp* expression in primary mouse OLs (Fig. 8A), supporting our hypothesis that EC1 and EC2 are critical to OL differentiation *in vitro*. A similar experiment with Plp1, another OL maker, corroborated this conclusion (Fig. S10). However, under the condition where the expression of *Plp1* was significantly downregulated, *Myrf* knockdown via CRISPRi silencing of the *Myrf* promoter, EC1, and EC2 did not consistently affect the morphological complexity of differentiating OLs (Fig. S10).

To test the importance of EC1 and EC2 for OL differentiation *in vivo*, we silenced EC1 and EC2 in the mouse brain by CRISPRi and examined its effect on OL differentiation by Gst-π and CC1 (mature OL markers allowing for easy cell counts^12^). Three plasmids were co-electroporated into subventricular zone (SVZ) neural stem cells (NSCs) of *Cnp*^*Cre/+*^ mice at P1 (postnatal day 1)^43,44^ (Fig. 8B). The first plasmid is a piggyBac-based one that expresses dCas9-KRAB and nuclear-targeted mCherry in a Cre-dependent manner. The second is a piggyBac-based plasmid that constitutively expresses sgRNAs. The third plasmid encodes hypBase, a hyperactive piggyBac transposase that integrates the first and second plasmids into the genome of SVZ NSCs^45^. Our electroporation protocol primarily targets SVZ NSCs on the striatum side^44^ (Fig. S11A). Since the expression of dCas9-KRAB and mCherry depends on the Cre recombinase, epigenome editing occurs mainly in Cnp-positive progenies of P1 SVZ NSCs, which are OPCs^46^. Electroporated brains were harvested at P28 for immunohistochemistry with mCherry and Gst-π. By blind cell count, we determined the fraction of mCherry-positive cells that are also positive for Gst-π (a late-stage OL marker). When dCas9-KRAB was targeted by Scr1, more than 87% of mCherry-positive cells were positive for Gst-π (Fig. 8C). It demonstrates that epigenome editing was specifically targeted to OL lineage cells, as we intended with the *Cnp1*^*Cre/+*^ system. When dCas9-KRAB was targeted to the *Myrf* promoter, EC1, EC2, or EC1&2, less than 12% of mCherry-positive cells were positive for Gst-π. The same results were obtained for CC1 (marking an earlier stage than Gst-π^12^; Fig. S11C,D). We conclude that EC1 and EC2 are crucial for OL differentiation *in vivo*.

## Discussion

Mapping enhancers to genes is a fundamental goal of modern biology. By combining recent advances in diverse fields into a coherent analysis pipeline, we have developed a simple yet powerful strategy that links enhancers to genes. This study illustrates its power by applying it to *Myrf*, a key OL gene that is indispensable for CNS myelination^10–12^. Three innovative features of our method enable a streamlined enhancer mapping for a gene of interest. First, it takes advantage of the transformative discovery from chromatin conformation capture studies that a gene and its enhancer tend to be found in the same TAD^13–15^. This provides strong spatial constraints on the possible locations of enhancers. Without TAD information, it is not clear where and how far to look in the genome for enhancers. TAD boundaries tend to be conserved between cell types and species^13^. Therefore, even if there is no Hi-C data for a particular cell type under study, one can still delineate the TAD by analyzing public Hi-C data^23^, as we did for *Myrf*. Second, we have derived a genome-wide map of 21324 putative OL enhancers. Without such map, the reduction of the enhancer search space by TAD analysis would not be really helpful because TADs are usually several hundred kilobases long. By comparing the genome-wide map of putative OL enhancers with the *Myrf* TAD, we uncovered 6 putative OL enhancers in the *Myrf* TAD, which are *Myrf* enhancer candidates because they are in the same TAD as *Myrf*. It is remarkable that the TAD information, together with our genome-wide map of putative OL enhancers, drastically reduces the search space for *Myrf* enhancers from the entire genome to just 6 loci, greatly facilitating the downstream CRISPRi analysis. Third, we have successfully used CRISPRi^19–22^, a cutting-edge epigenome editing technique, to interrogate enhancer candidates, finding two *Myrf* enhancers that are essential for OL development (EC1 and EC2). By inactivating enhancers in the genomic context, CRISPRi allows one to determine whether an enhancer candidate governs the expression of a given gene in the genomic context. As shown by this study and others, CRISPRi is exquisitely specific, even in promoter upstream regions and gene bodies, enabling the discovery of promoter-distal enhancers as well as those in promoter upstream regions and gene bodies. In sum, the combination of TAD, a cell type-specific genome-wide map of putative enhancers, and CRISPRi would significantly accelerate mapping enhancers to target genes. For cell type-specific genome-wide maps of putative enhancers, the single-cell ATAC-seq data and the NIH Roadmap Epigenomics Project data that we analyzed are good enough to cover most cell types. Of course, one can add publicly available cell type-specific genomic data to further strengthen such maps, as we did for OL lineage cells.

Encouraged by the positive results for *Myrf*, we are currently applying our new method to other OL genes and other cell types. For example, our ongoing study on *Olig2*, a gene essential for oligodendrogenesis, found that the *Olig2* TAD is well defined and conserved between cell types and species, leading to a small number of *Olig2* enhancer candidates for CRISPRi analysis. The same was also true for key myelin components (*Cnp*, *Mbp*, *Plp1*), critical OL transcriptional regulators (*Olig1*, *Zeb2*, *Sox10*, *Nkx2.2*, *Sox2*, *Tcf7l2*), and OL differentiation inhibitors that are potential therapeutic targets for remyelination therapies (*Gpr17* and *Gpr37*).

If an enhancer is fully redundant with another, it may elude discovery because its inactivation would not show any effect. It is not clear how such redundancy would affect CRISPRi. In this regard, a previous analysis of HS2, an enhancer in the β-globin locus control region, by dCas9-KRAB is informative. Genetic deletion of HS2 in mice had only a mild effect on the expression of β-globin genes despite it being a strong enhancer^47^. In contrast, epigenome editing analysis of HS2 in human cells revealed that its inactivation by dCas9-KRAB significantly downregulates the expression of several β-globin genes^20^. Even though it is not possible to directly compare the two studies because of several differences (*e.g*., one study in mice while the other in a human cell line), the human cell line study suggests that CRISPRi may be able to overcome the redundancy issue. On the other hand, CRISPRi can easily be multiplexed, and one potential way to overcome enhancer redundancy is to silence multiple enhancer candidates simultaneously by co-expressing different sgRNAs, as we did for EC1 and EC2. Alternatively, one may supplement CRISPRi with CRISPRa, an epigenome editing technique that activates enhancers and upregulates target genes in the genomic context^22,48^, which may suffer less from enhancer redundancy than CRISPRi.

## Methods

### Reagents

A mouse *Myrf* cDNA that encodes the 1139 amino acid-long isoform was kindly provided by Dr. Ben Emery. The *Myrf* cDNA was cloned into pcDNA3. To generate epigenome editing constructs, we amplified dCas9-KRAB from pHAGE EF1α dCas9-KRAB (Addgene #50919) by PCR and replaced the Cre portion of pCAG-Cre (Addgene #13775) with it and an IRES (internal ribosome entry site)-tdTomato cassette. For SVZ electroporation, the piggyBac ITRs (inverted terminal repeat) were inserted, and tdTomato replaced with nuclear-targeted mCherry (Addgene #20972). To generate sgRNA expression vectors, the *EF-1α* promoter of pSBbi-RN (Addgene #60519) was replaced by the sgRNA scaffold taken from lentiCRISPR v2 (Addgene #52961). For the SVZ electroporation of sgRNAs, the content of PB-CA (Addgene #20960) was replaced by the sgRNA scaffold. Rffl, a Myrf luciferase reporter, was generated by cloning a rat genomic fragment (rn4 chr10:71034166–71034749) into pGL3-promoter (Promega). The sequence information of all constructs was verified by Sanger sequencing, and protein expression was confirmed by Western blot. The Myrf antibody was generously provided by Michael Wegner^5^. The sources of the commercial antibodies used for the study are as follows: FLAG (Sigma F1804) RFP (Rockland 600-901-379), MBP (Millipore MAB386), CC1 (Millipore OP80), Gst-π (MBL 311), donkey anti-rat IgG, Alexa Fluor 594 (Invitrogen A21209), donkey anti-rabbit IgG, Alexa Fluor 488 (Invitrogen A21206), donkey anti-mouse IgG, Alexa Fluor 488 (Invitrogen A21202), and donkey anti-chicken IgY, Alexa Fluor 594 (Jackson 703-585-155).

### Animal procedures, tissue harvest, and cell culture

The current study was conducted in strict accordance with the protocol (approved protocol number #NA-Park2) approved by the Institutional Animal Care and Use Committee of SUNY Buffalo, which is licensed by the National Institutes of Health Office of Laboratory Animal Welfare (animal welfare assurance number: D16-00231). OPCs were purified from mouse pups of P7 ~ P9 by immunopanning^36^. The original immunopanning protocol for mouse OPCs^35^ did not work well in our hands. Instead, we found that the immunopanning protocol for rat OPCs works well for mouse OPCs, and this is why we used it to purify mouse OPCs. Primary mouse OPCs and Oli-neu cells^39^ were kept in a proliferative condition by supplementing the Sato media^36^ with PDGF (10 μg/mL), NT3 (1 μg/mL), CNTF (10 μg/mL), and NeuroCult™ SM1 Neuronal Supplement. They were maintained in a humidified 8% CO_2_ incubator at 37 °C. Transient transfection was performed using Lipofectamine 2000 as per the manufacturer’s instructions.

### Genome-wide map of putative OL enhancers

The OL ChIP-seq data were downloaded from the Sequence Read Archive^49^. All the OL ChIP-seq data are from cultured rat OL lineage cells, and their accession numbers are as follows: Brg1 (GSM1040154, GSM1040155), Chd7 (GSM1869162), H3K27ac (GSM1040159, GSM1040160, GSM1040161), H3K4me3 (GSM1040162, GSM1040163, GSM1040164), Olig2 (GSM1040156, GSM1040157, GSM1040158), Sox10 (GSM1869163, GSM1577133, GSM1577134), Tcf7l2 (GSM1587566, GSM1587567, GSM1587568). The Myrf ChIP-seq data were downloaded from the journal website (https://journals.plos.org/plosbiology/article?id = 10.1371/journal.pbio.1001625). ChIP-seq reads were mapped to rn4 by Bowtie^50^, and peaks called by MACS2^51^. The genome-wide map of 21324 putative OL enhancers were derived in rn4 and mapped to mm9 by liftOver using default options. For comparison with the Roadmap Epigenomics Project data, the six *Myrf* enhancer candidates were mapped to hg19 by liftOver using default options.

For the statistical analysis of OL H3K27ac profiles, the three OL H3K27ac ChIP-seq data (GSM1040159, GSM1040160, and GSM1040161) were merged, and the merged H3K27ac profile analyzed by the cumulative binomial distribution function. For a hypothetical valley (V) flanked by the left and right shoulders (LS and RS), we count the number of reads mapped to each region. VR: # of reads for V, LSR: # of reads for LS, and RSR: # of reads for RS. Also we define their sizes in base pairs (bps). VS: size of V, LSS: size of LS, and RSS: size of RS. Under the null model, H3K27ac ChIP-seq reads would be distributed randomly (*i.e*., uniformly). We quantify the deviation of the observed read distribution around the valley from the null model by the cumulative binomial distribution function as follows. For the left shoulder, compute the cumulative binomial probability of observing VR or less reads for V given the total number of reads being VR + LSR under the probability of VS/(VS + LSS). Since the sizes of valleys and shoulders are not pre-defined, we allow the valley to take any value from 250, 300, 350, and 400 bps. Shoulders are allowed to take any value from 300, 400, and 500 bps. From these 12 combinations, we take the lowest *p* value as a score for the valley. We repeat the same calculation for the right shoulder. Then we take the greater of the two *p* values as the final score for the valley. Once this computation was finished for the entire genome, the valleys were ranked by their scores. We leniently picked the top 6939 valleys as putative OL enhancers by cross-examining the valleys’ scores with their distributions of H3K27ac ChIP-seq reads.

### ATAC-seq and the NIH roadmap epigenomics project data

The ATAC-seq data were downloaded from the Shendure laboratory website (http://atlas.gs.washington.edu/mouse-atac). The NIH Roadmap Epigenomics Project data were visualized by the WASHU Epigenome Browswer.

### Luciferase assay-based epigenome editing analysis

Single guide RNAs (sgRNAs) were designed by using a web service from the Zhang laboratory (zlab.bio/guide-design-resources). For epigenome editing, dCas9-KRAB, gRNAs, Rffl, and pRL-TK (an internal control for ratiometric luciferase analysis, Promega) were transfected into primary mouse OPCs, which were then cultured for 3 days in the differentiation condition. The reporter activity of Rffl relative to that of pRL-TK was determined by the Promega dual luciferase reporter assay kit, as per the manufacturer’s instructions.

### RT-qPCR

Total RNA was purified using Trizol (Thermo Fisher Scientific #15596026), and cDNA synthesized by the SuperScript First-Strand kit (Invitrogen #11904-018). Quantitative PCR was performed by C1000 Touch thermal cycler with the CFX384 optical reaction module (Bio-rad). The expression level of a gene was normalized to that of *Gapdh*. Each PCR reaction contained 2 µL of cDNA, 5 µL of the iTaq Universal SYBR Green Supermix (Bio-rad #1725124), and 500 nM of forward and reverse primers. The primer sequences are shown in Table 1.

**Table 1:** Primer sequences for RT-qPCR.

*Myrf*	Forward	CATTGTGCGGGCCTCTAACCC
	Reverse	CCTCATCTGGCCGGTCGG
*Dagla*	Forward	GCCGCACCTTCGTCAAGC
	Reverse	GACCAGCTGGTGGCCTGAC
*Syt7*	Forward	CCACTGGTGTCAGCGCAAACTG
	Reverse	GCTTTCTTCTCACCGCGCCC
*Sdhaf2*	Forward	CCTTGATCCCGACGCTGGC
	Reverse	GAGTCTGTTGGGCTGTCACCTCTG
*Cpsf7*	Forward	CCCAAGAGGGGGAATACCTCCAC
	Reverse	GGGCTTATCCACACGAGCAGATGAG
*Tmem258*	Forward	CCTGGTTCTTCGTTTACGAGGTCAC
	Reverse	GGAGGAAGAGGACTCCAAAGCC
*Fads1*	Forward	CCCCTCTTCTTCGCCCTG
	Reverse	GGGGTCCGATGAGGAAGAAGTAC
*Gapdh*	Forward	GGTGAAGGTCGGTGTGAACGG
	Reverse	CTGGAACATGTAGACCATGTAGTTGAGG

### *In vitro* differentiation assay of primary OPCs

DNA plasmids that express dCas9-KRAB and sgRNAs were transfected into primary mouse OPCs using Lipofectamine 2000. Transfected OPCs were kept in the differentiation condition for 3 days. Cells were fixed with 4% formaldehyde and permeabilized with 0.1% Triton X-100. Upon blocking with 1% BSA, they were incubated with primary antibodies diluted in the blocking buffer at 4 °C overnight, followed by incubation with fluorochrome-conjugated secondary antibodies. Nuclei were stained with Hoechst 33342 (Invitrogen). Fluorescence was visualized with a Leica DMi8 microscope with an ORCA-Flash4.0 sCMOS camera. Images were taken in a blind manner. The signal from each fluorescence channel (Hoechst, GFP, and RFP) was quantified by CellProfiler for an objective quantitative analysis.

### SVZ electroporation

P1 *Cnp*^*Cre/+*^ pups were anesthetized by hypothermia. Five µl of DNA solution, including dCas9-KRAB, gRNA, the hyperactive piggyBac transposase, and 0.05% of Fast Green Dye for visualization, was injected into brain ventricles with a fine-tipped micropipette. After injection, electroporation paddles (wet with saline) were placed on the sides of the pup head, and five 40 V, 50 ms square pulses at a frequency of 60 Hz, were delivered. Our electroporation protocol mainly targets SVZ NSCs on the striatum side. The pup was allowed to recover on a heating pad. Electroporated pups were euthanized at P28. The brain tissue was fixed in 4% paraformaldehyde in PBS for 2 days, which was followed by an incubation in 30% sucrose for 1~2 days. The brain tissue was cut in 10 µm thickness. Brain sections were washed in PBS, permeabilized in 0.3% Trition-X100, and blocked in 5% FBS, 0.3~0.6% Triton-X100 in PBS. The sections were incubated with primary antibodies, washed in PBS, incubated with secondary antibodies, and washed in PBS. They were mounted in a mounting medium (VectaShield H-1000). Fluorescence was visualized with the Leica DMi8 microscope in a blind manner. Cells were counted in a blind manner by two independent researchers for robust statistical analysis.

## Supplementary information

Supplementary Information

## Data Availability

The datasets generated and/or analysed during the current study are available from the corresponding author on reasonable request.
