# Supplementary material for: A principled strategy for mapping enhancers to genes
Source: Sci Rep. 2019 Jul 30;9:11043. doi: 10.1038/s41598-019-47521-w (PMC6667464; doi:10.1038/s41598-019-47521-w)
Supplement: Supplementary file 1 — Supplementary Information [file 41598_2019_47521_MOESM1_ESM.pdf]

Supplementary Information

A principled strategy for mapping enhancers to genes

Dongkyeong Kim[1], Hongjoo An[1], Randall S. Shearer[1], Mohamed Sharif[1], Chuandong Fan[1], Jin-ok Choi[1], Sun Ryu[1], and Yungki Park[1,*]

[1]Hunter James Kelly Research Institute, Department of Biochemistry, Jacobs School of Medicine and Biomedical Sciences, University at Buffalo, Buffalo, NY 14203, USA

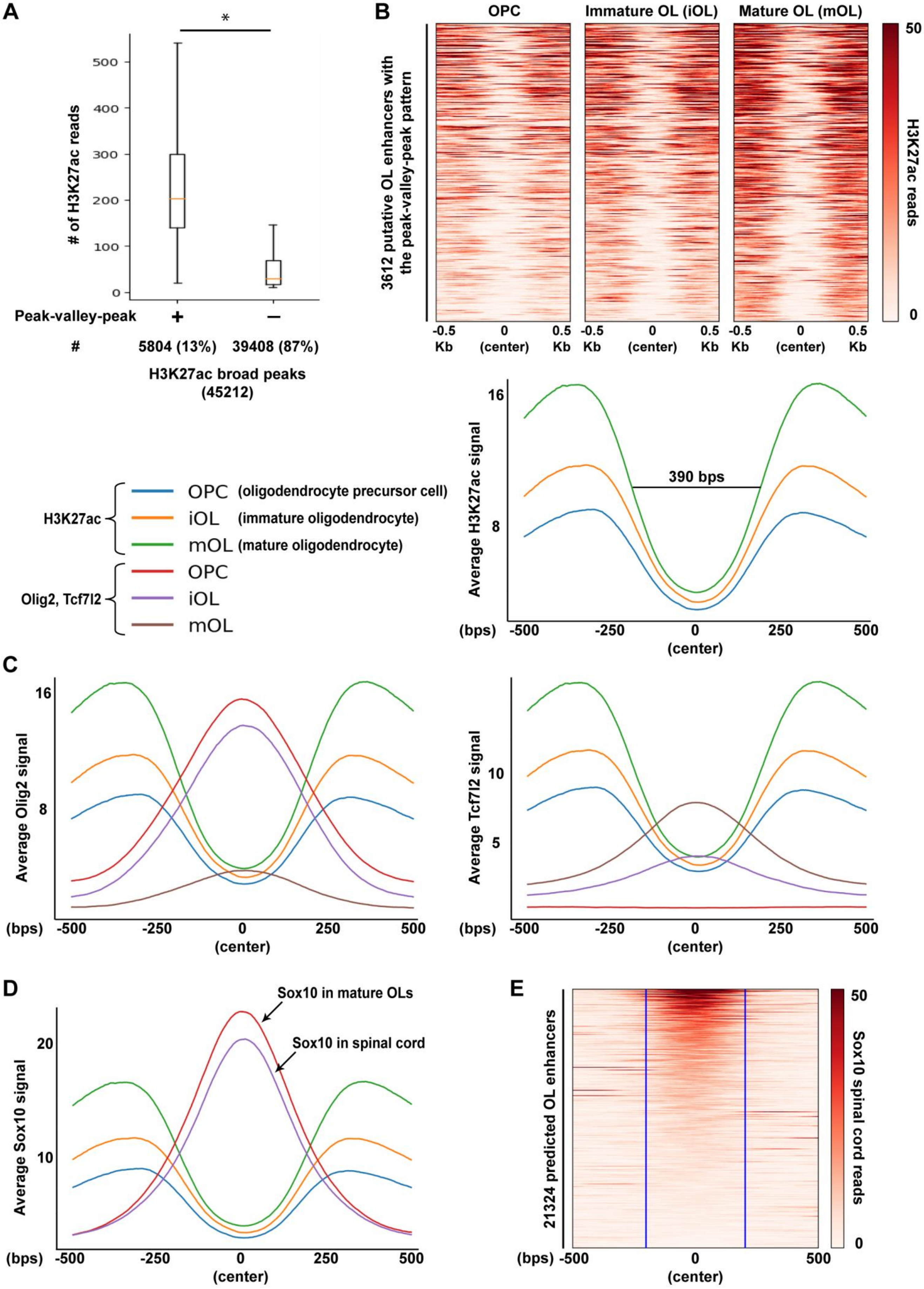

Figure S1. Characterization of the putative OL enhancers

(A) The 45212 broad peaks in the OL H3K27ac ChIP-seq data were divided into two groups depending on the presence of the peak-valley-peak pattern. About 13% (=5804/45212) have at least one incidence of the peak-valley-peak configuration. The 5804 H3K27ac broad peaks have a significantly higher level of H3K27ac signals than the rest. *p value ≈ 0 by the Mann–Whitney–Wilcoxon test. (B) About 17% (=3612/21324) of the putative OL enhancers in the final set is overlaid with the H3K27ac peak-valley-peak pattern. H3K27ac ChIP-seq signals for the 3612 putative OL enhancers and their average profiles are shown. The size of the putative OL enhancers was defined by the distance between the half-points of the flanking H3K27ac ChIP-seq peaks, which is 390 base pairs. (C&D) Nucleosome depletion for the 3612 putative OL enhancers is likely due to the competitive DNA binding of transcription factors because ChIP-seq peaks for Olig2, Tcf7l2, and Sox10 fall within the 390 base pair-long region. (E) Alignment of Sox10 spinal cord ChIP-seq reads against the 21324 putative OL enhancers in the final set. The blue lines demark the boundaries of the putative OL enhancers.

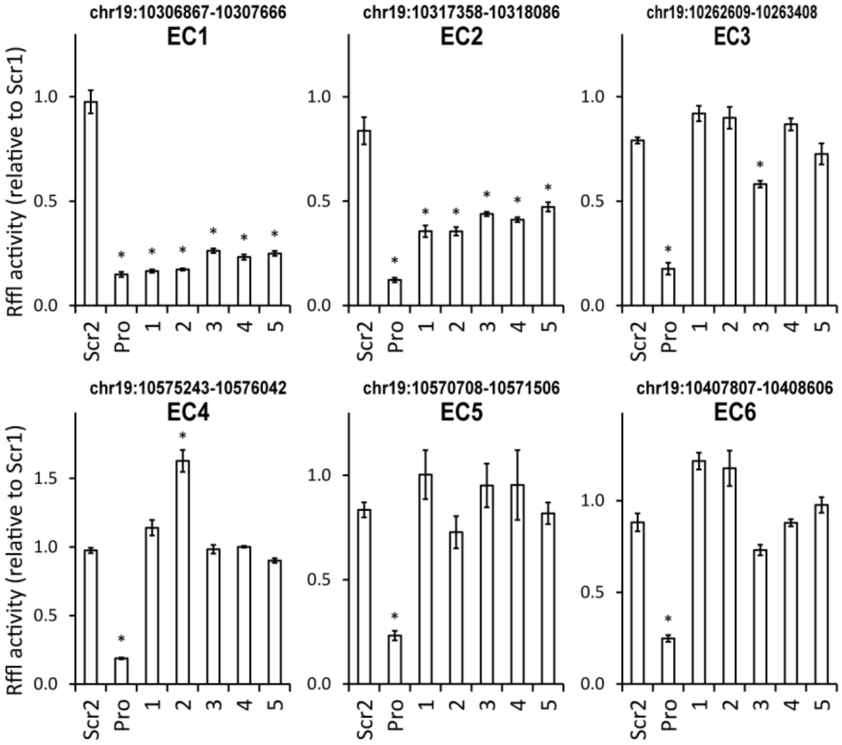

Figure S2. Interrogation of the 6 *Myrf* enhancer candidates by CRISPRi in Oli-neu cells

For each sgRNA, the mean and standard error are shown. *p value < 1.2×10^{-2} by two-sided unpaired Student's t test corrected by the Bonferroni procedure (comparison with Scr2). Scr1 and Scr2 are two non-targeting negative control sgRNAs. The number of biological replicates is 4 for EC1, EC2, and EC3 and 3 for EC4, EC5, and EC6.

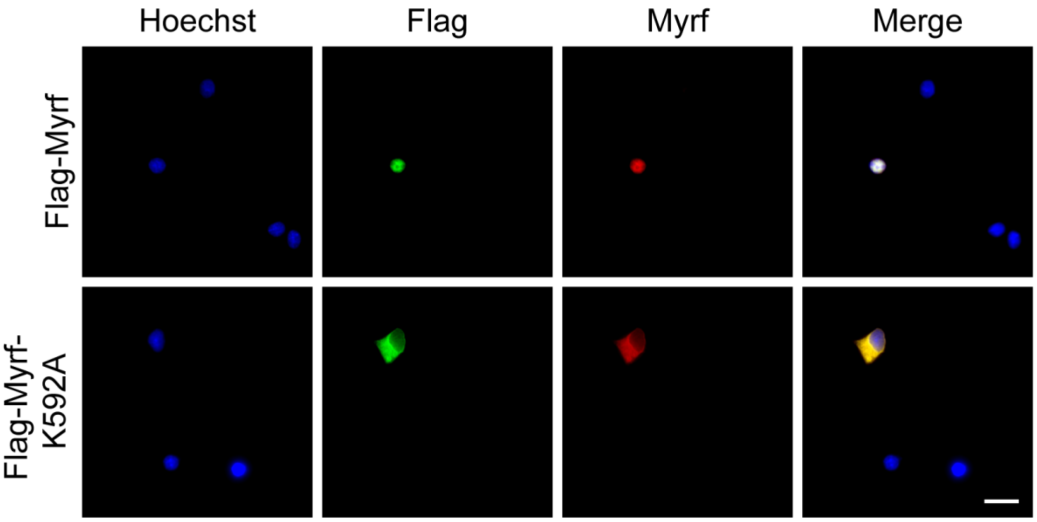

Figure S3. Validating the Myrf antibody by immunofluorescence in Oli-neu cells
The epitope of this Myrf antibody lies in the N-terminal portion of Myrf, and thus this antibody recognizes Myrf N-terminal fragment and full-length Myrf. Flag-tagged Myrf constructs were transfected into Oli-neu cells, and the immunofluorescence of the Myrf antibody was compared with that of the Flag antibody (Sigma F1804). Scale bar, 20 µm.

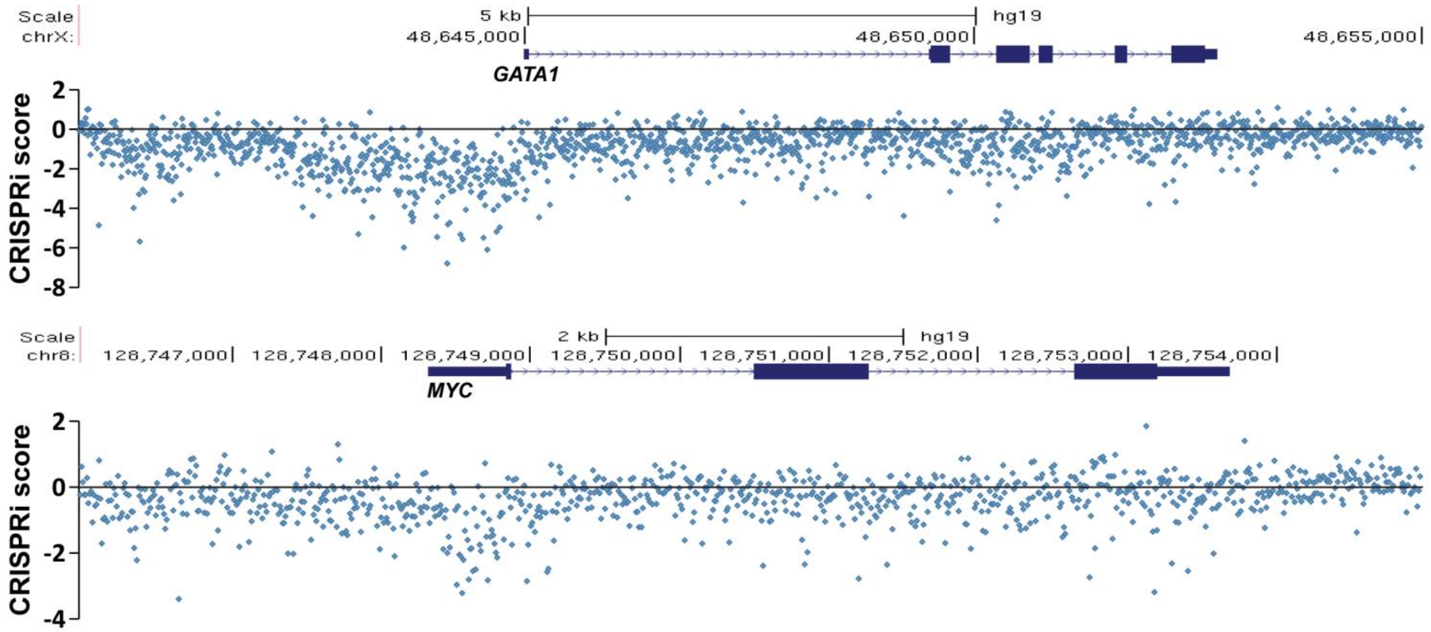

Figure S4. The CRISPRi screen results for *GATA1* and *MYC* (*Science* (2016) 354:769)
In this screen, the CRISPRi score 0 means no effect on the expression of the respective gene. Negative scores mean negative expression. These plots were generated by using the data downloaded from the journal website (http://science.sciencemag.org/content/354/6313/769.long; *Science* (2016) 354:769). It reveals that epigenome editing by dCas9-KRAB is highly specific, even in promoter upstream regions and gene bodies.

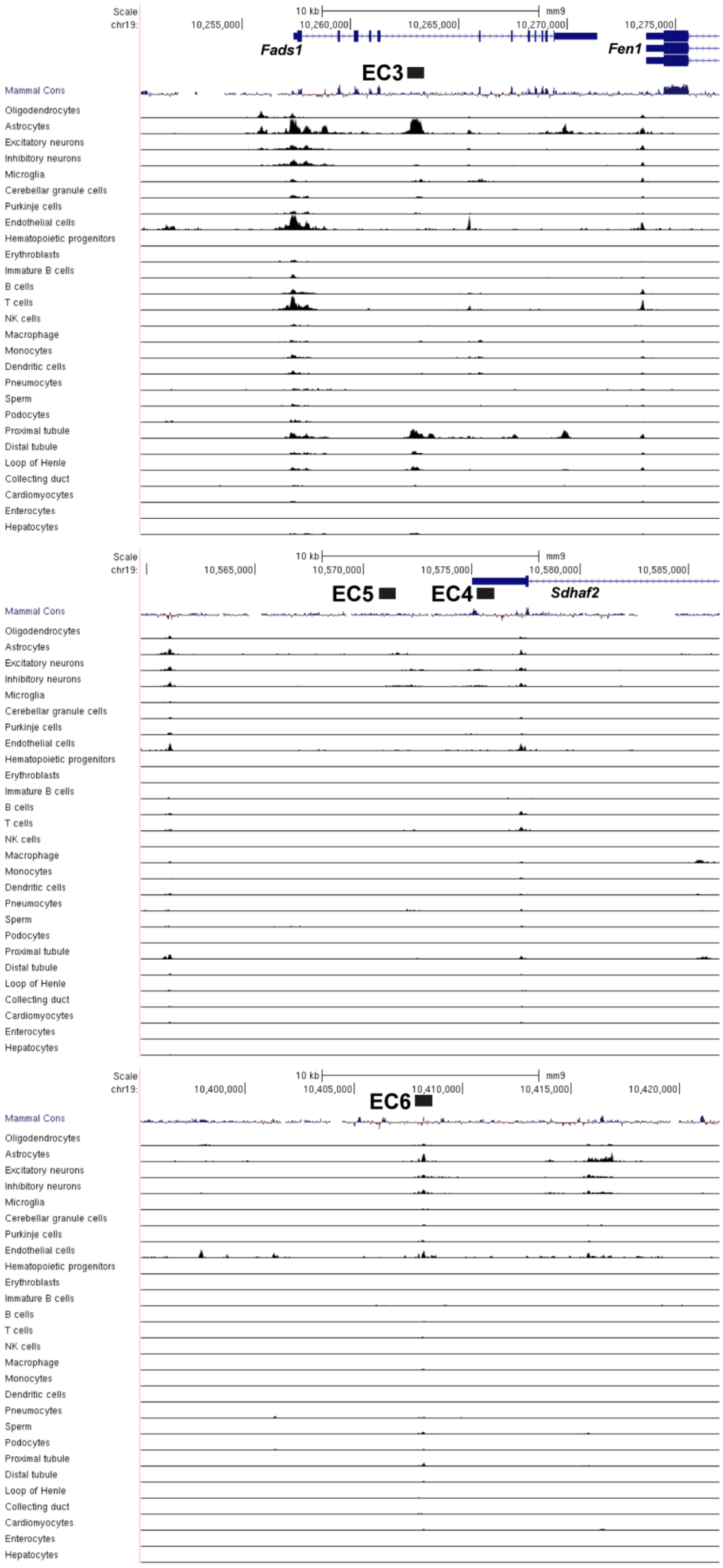

Figure S5. Mouse single-cell ATAC-seq data for the 4 negative *Myrf* enhancer candidates. These data were downloaded from http://atlas.gs.washington.edu/mouse-atac/. Please refer to the original publication (*Cell* (2018) 174:1309) for the detail.

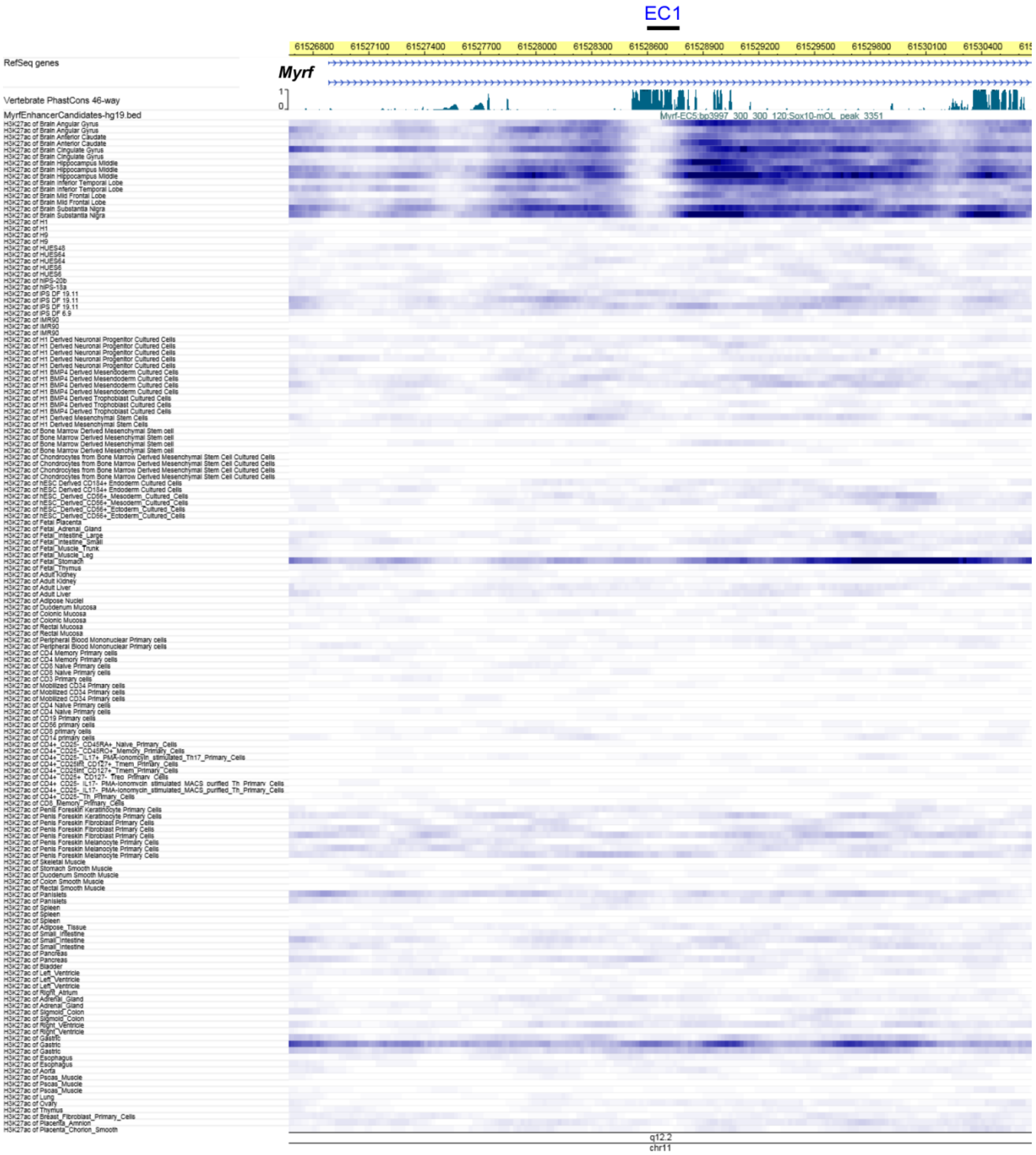
EC1
61526800
61527100
61527400
61527700
61528000
61528300
61528600
61528900
61529200
61529500
61529800
61530100
61530400
RefSeq genes
Myrf
Vertebrate PhastCons 46-way
MyrfEnhancerCandidates-hg19.bed
Myrf-EC5:bp3997_300_300_120:Sox10-mOL_peak_3351
q12.2
chr11

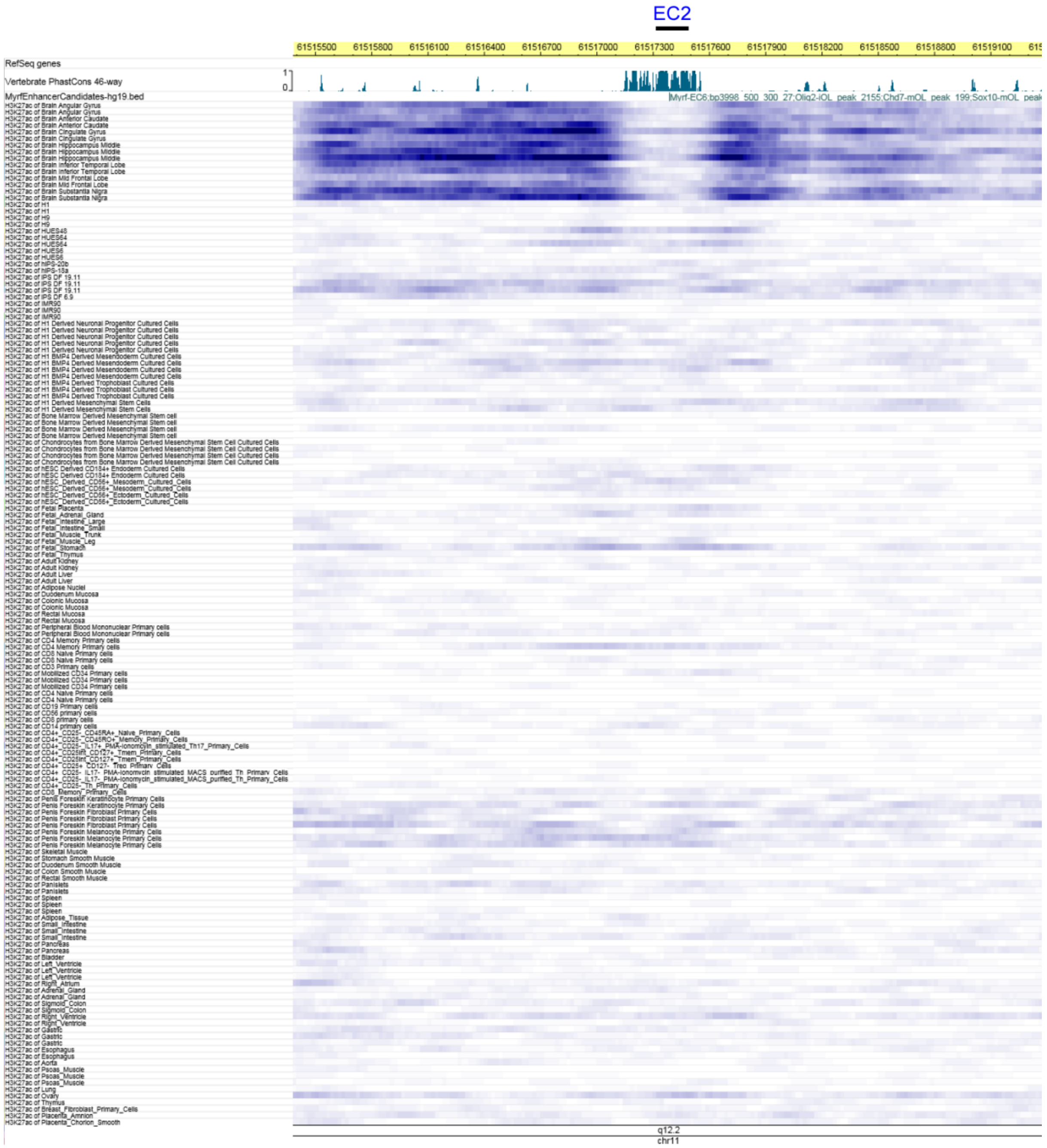

Figure S6. Complete NIH Roadmap Epigenomics Project H3K27ac ChIP-seq data for EC1 and EC2

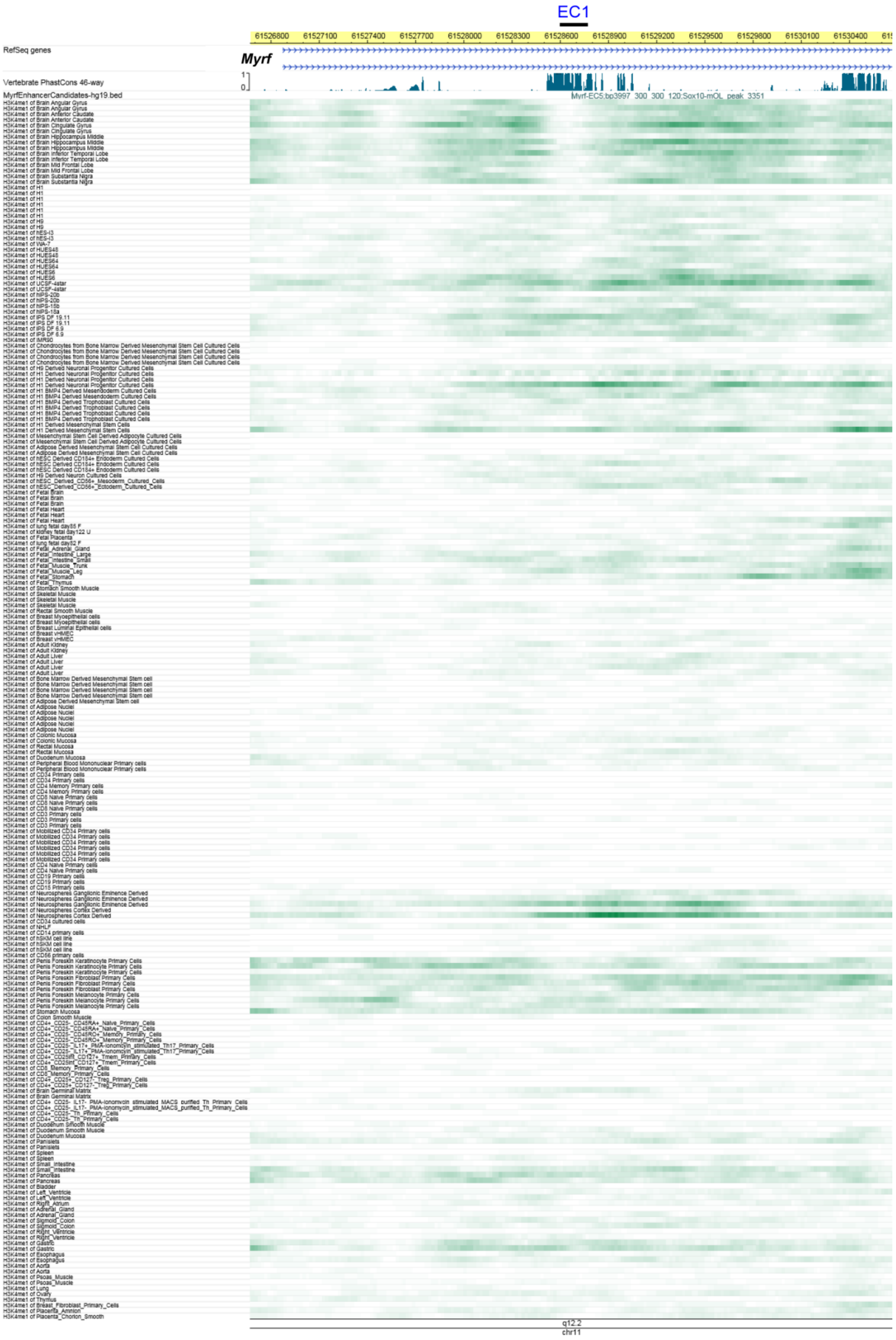
EC1
61526800 61527100 61527400 61527700 61528000 61528300 61528600 61528900 61529200 61529500 61529800 61530100 61530400
RefSeq genes
Myrf
Vertebrate PhastCons 46-way
MyrfEnhancerCandidates-hg19.bed
Myrf-EC5;bp3997_300_300_120;Sox10-mOL_peak_3351
q12.2
chr11

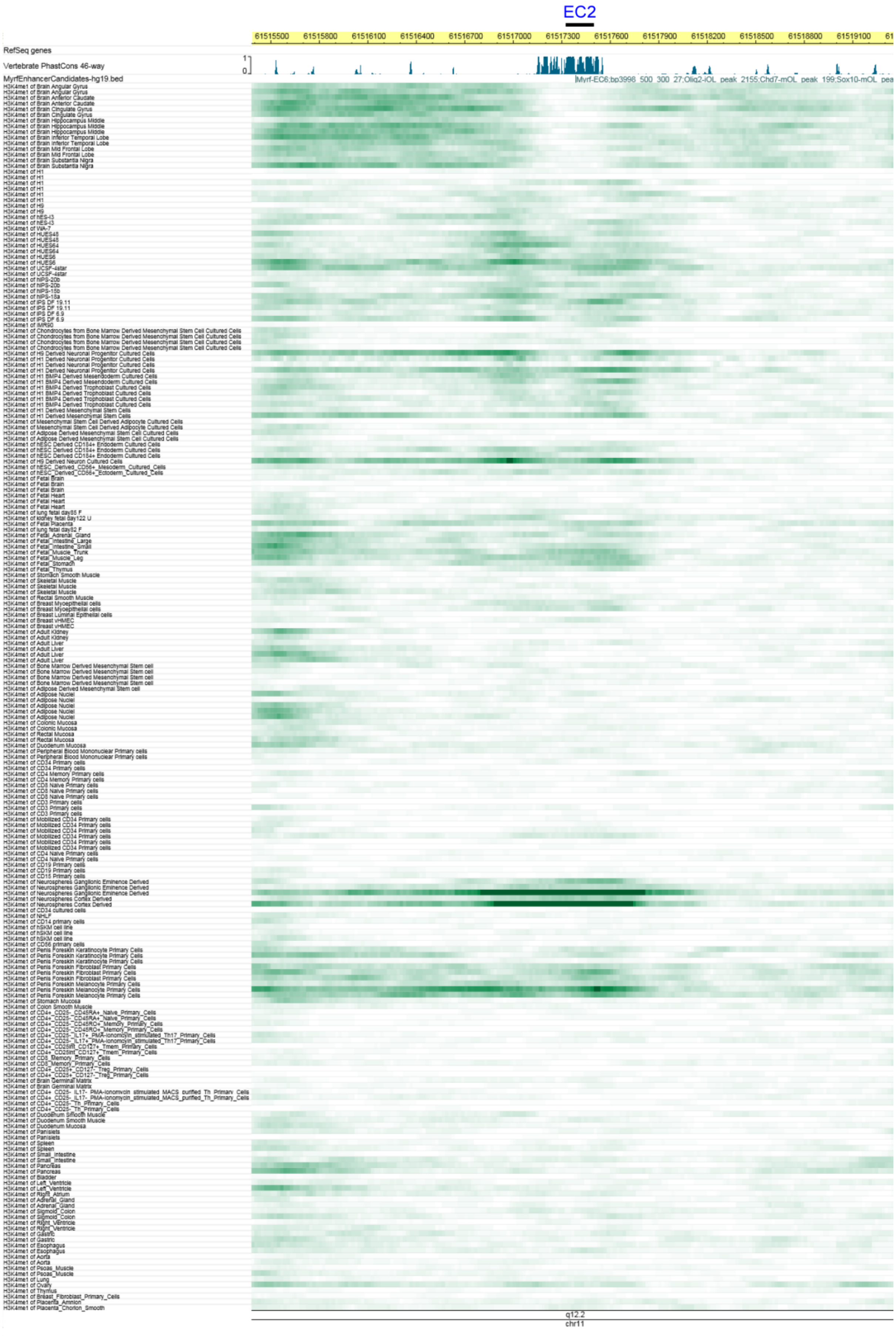

Figure S7. Complete NIH Roadmap Epigenomics Project H3K4me1 ChIP-seq data for EC1 and EC2

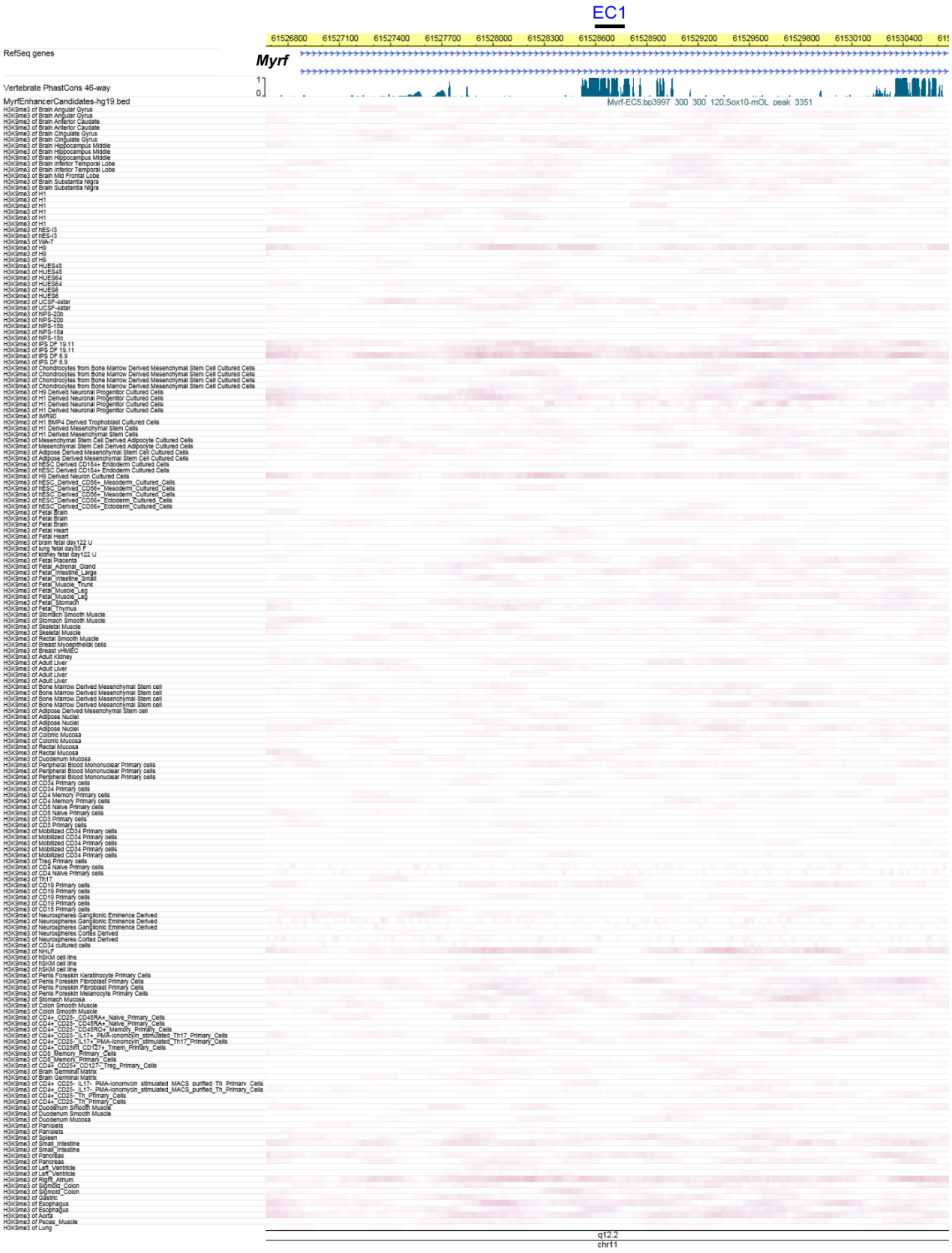
EC1
61526800
61527100
61527400
61527700
61528000
61528300
61528600
61528900
61529200
61529500
61529800
61530100
61530400
RefSeq genes
Myrf
Vertebrate PhastCons 46-way
1
0
MyrfEnhancerCandidates-hg19.bed
Myrf-EC5;bp3997_300_300_120;Sox10-mOL_peak_3351
q12.2
chr11

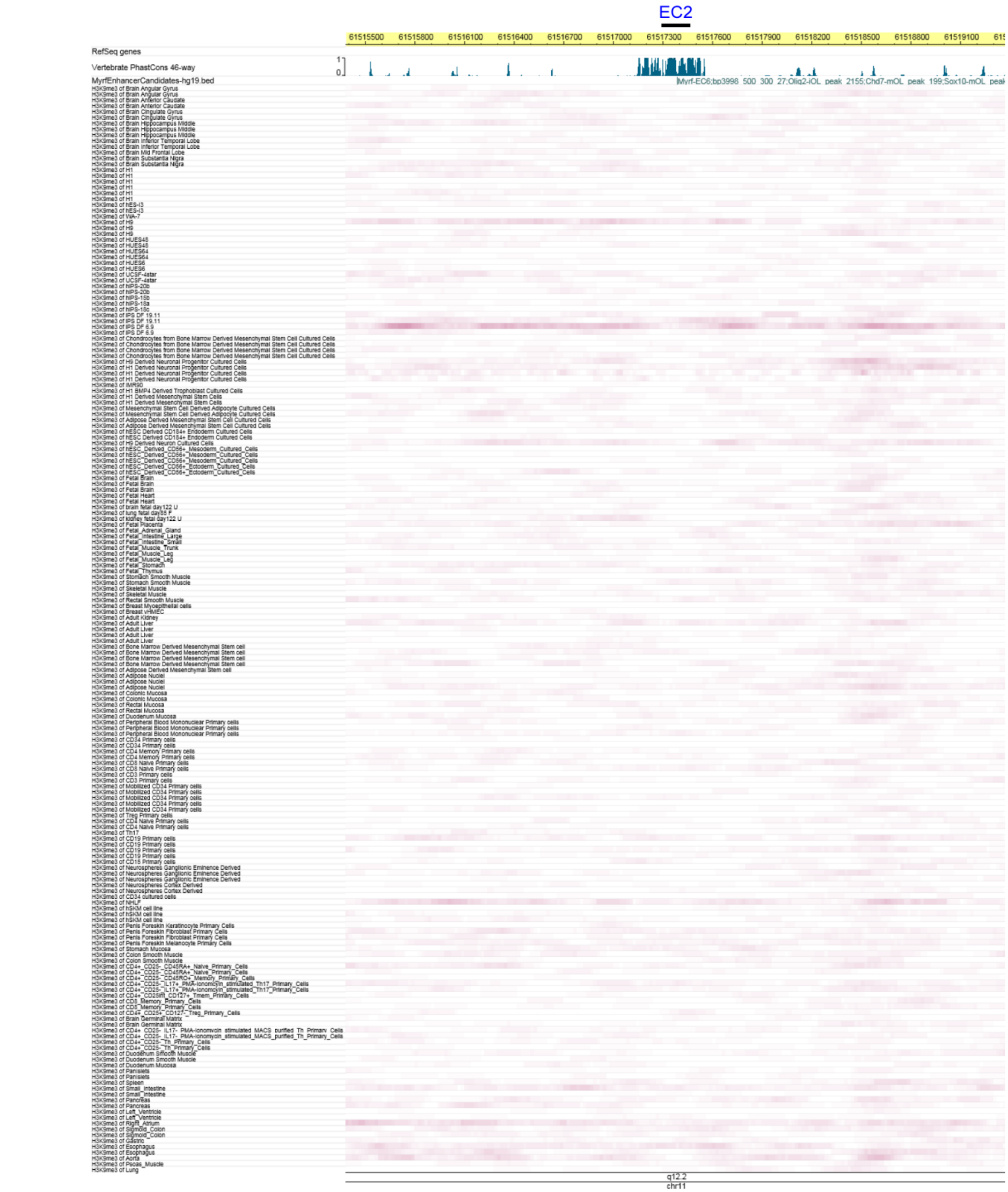

Figure S8. Complete NIH Roadmap Epigenomics Project H3K9me3 ChIP-seq data for EC1 and EC2

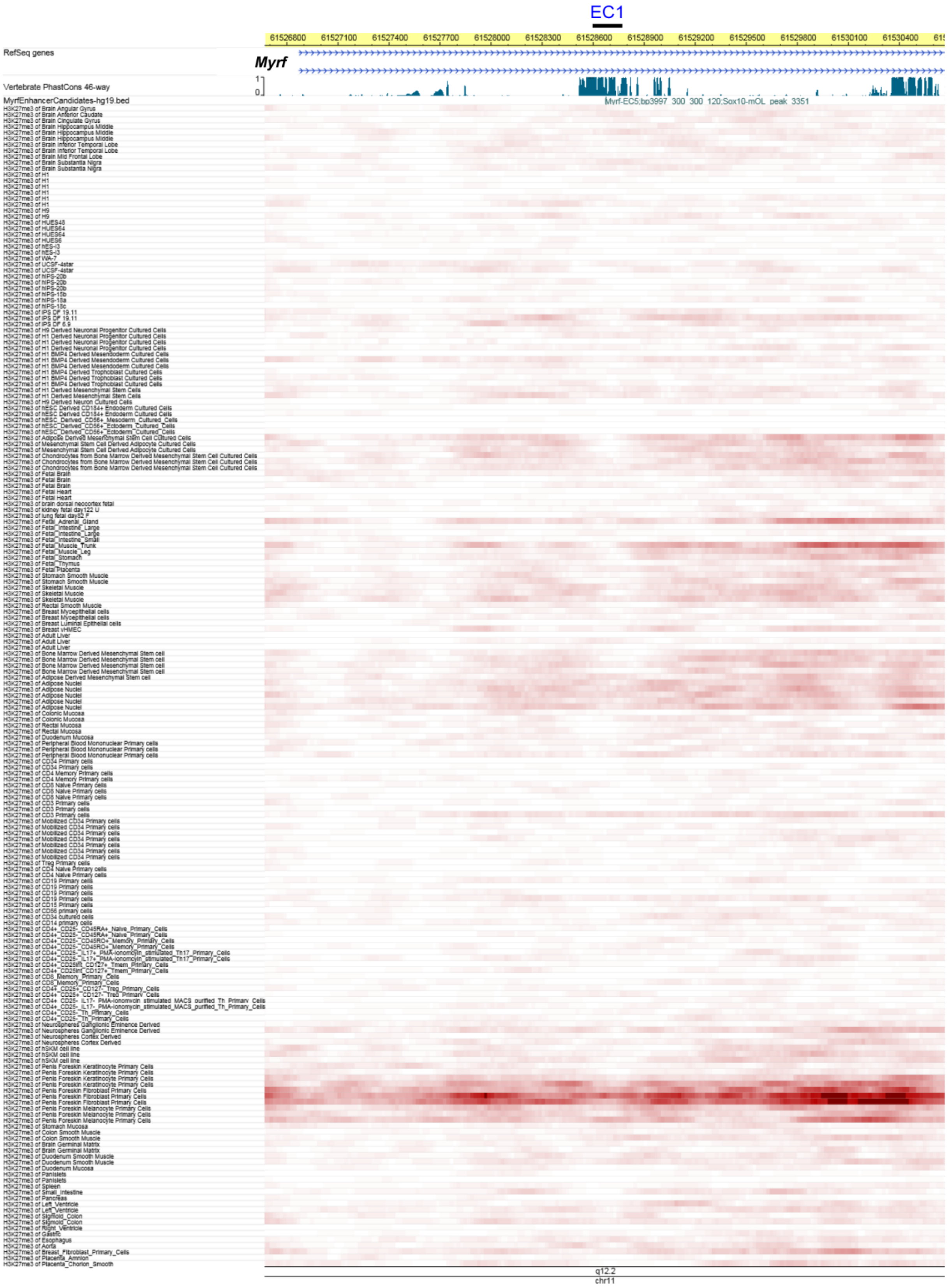
EC1
61526800
61527100
61527400
61527700
61528000
61528300
61528600
61528900
61529200
61529500
61529800
61530100
61530400
RefSeq genes
Myrf
Vertebrate PhastCons 46-way
1
0
MyrfEnhancerCandidates-hg19.bed
Myrf-EC5;bp3997_300_300_120;Sox10-mOL_peak_3351
q12.2
chr11

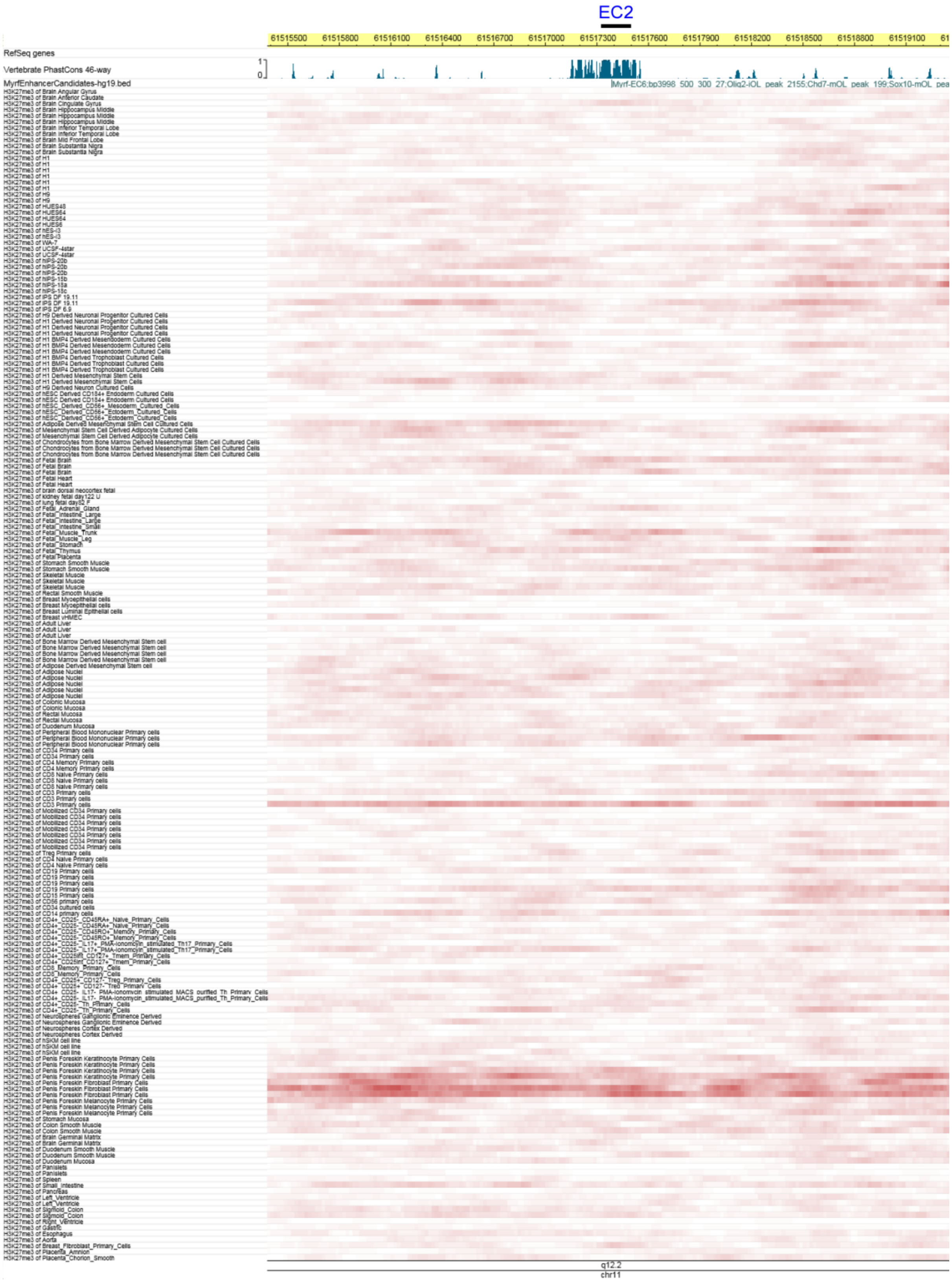

Figure S9. Complete NIH Roadmap Epigenomics Project H3K27me3 ChIP-seq data for EC1 and EC2

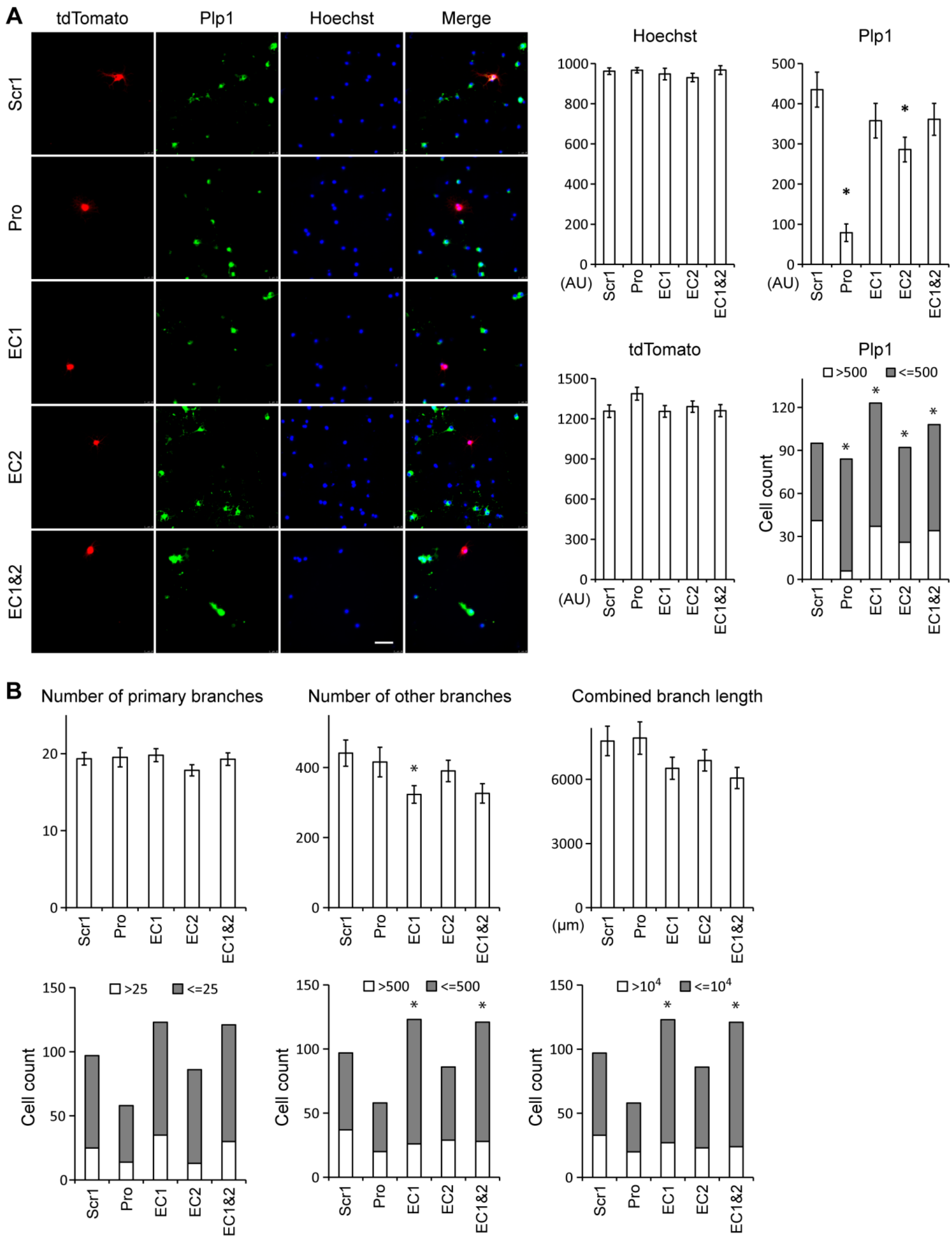

Figure S10. The impact of CRISPRi silencing EC1 and EC2 on OL differentiation, as assessed by *Plp1* expression and morphological maturation

(A) Immunostaining showed that targeting dCas9-KRAB to the *Myrf* promoter (Pro, by G5 in **Figure 5A**), EC1 (by 5 in **Figure 5B**), EC2 (by 4 in **Figure 5B**), or EC1&2 (by 5 and 4 in **Figure 5B**, respectively) decreases *Plp1* expression in primary mouse OLs. Scale bar, 50 μm. The signal from each fluorescence channel was

quantified for individual cells by CellProfiler. AU: arbitrary unit. The distribution of Plp1 signals for Pro and EC2 is significantly shifted downward compared to that for Scr1. *p value $< 2.5 \times 10^{-2}$ by two-sided unpaired Student's t test corrected by the Bonferroni procedure. The distribution of Plp1 signals for EC1 and EC1&2 is also shifted downward, but not statistically significantly. The cumulative binomial distribution function revealed that CRISPRi silencing of EC1 and/or EC2 impairs a high level expression of *Plp1*, as defined by > 500 AU. *p value $< 2.4 \times 10^{-2}$ after correction by the Bonferroni procedure. (B) The same samples were analyzed to determine the impact of silencing EC1 and/or EC2 on the morphological maturation of differentiating OLs. Other branches mean all branches except for primary ones (*i.e.*, secondary, tertiary, and so on). Comparison with Scr1 showed that CRISPRi silencing of EC1 significantly decreases the number of other branches. *p value $< 3.1 \times 10^{-2}$ by two-sided unpaired Student's t test corrected by the Bonferroni procedure. Since there is a downward trend for EC1 and EC1&2 for the number of other branches and combined branch length, we also analyzed the samples by the cumulative binomial distribution function. It revealed that CRISPRi silencing of EC1 and EC1&2 significantly decreases the number of other branches and combined branch length. *p value $< 9.8 \times 10^{-3}$ after correction by the Bonferroni procedure. Importantly, however, we did not observe any effect in the Pro and EC2 samples, even though they exhibited a much bigger drop in *Plp1* expression (panel A). Thus, the morphology results are inconclusive, and we are unable to reject the null hypothesis that *Myrf* knockdown by CRISPRi silencing EC1 and EC2 does not impact the morphological development of differentiating OLs.

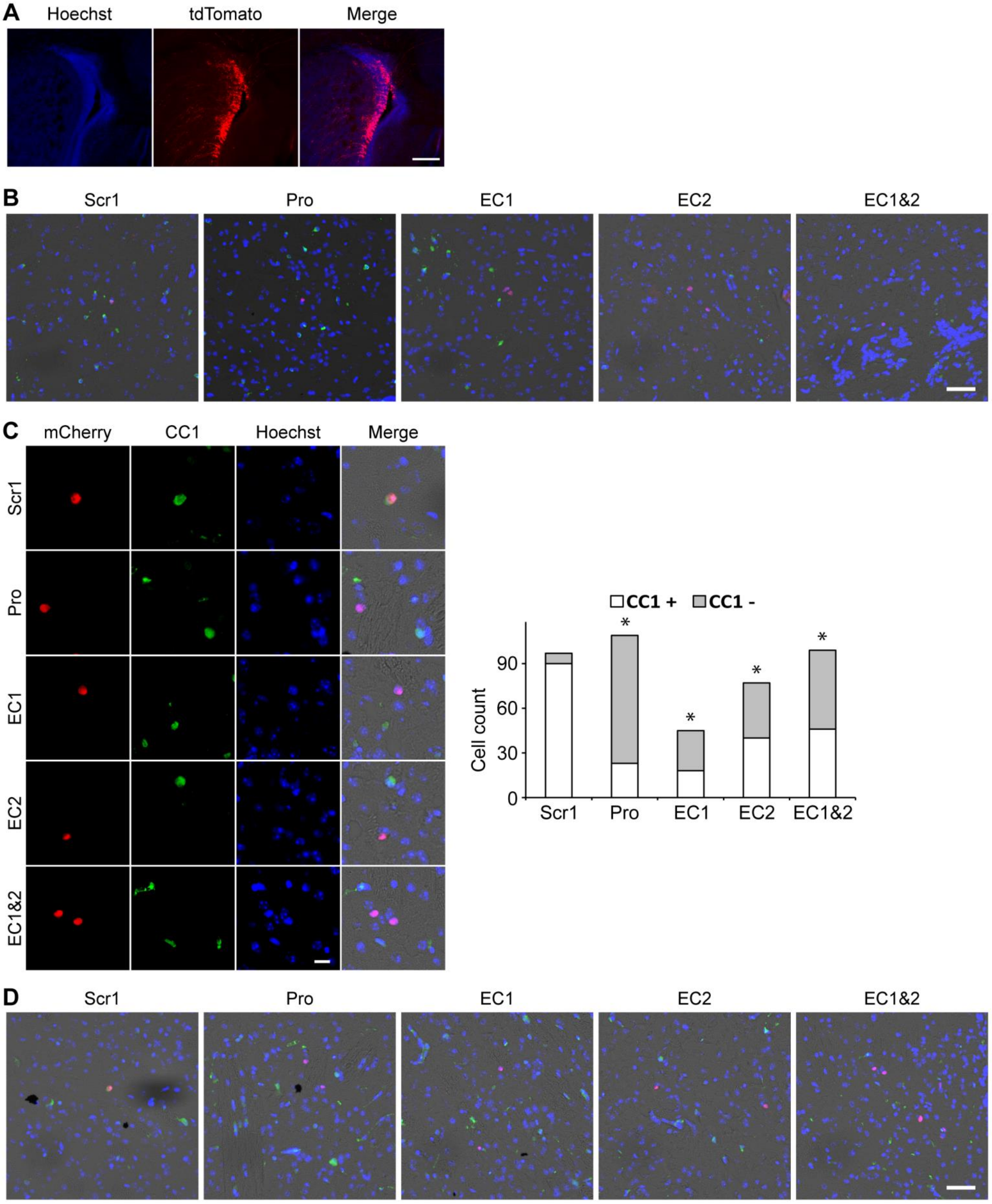

Figure S11. EC1 and EC2 are required for OL differentiation in the mouse brain

(A) To demonstrate SVZ electroporation, a piggyBac-based tdTomato plasmid was electroporated into SVZ NSCs in the lateral ventricle of a P2 C57BL/6 mouse. The brain was harvested at P4 and stained for Hoechst. SVZ NSCs in the striatum side were successfully electroporated, as shown by tdTomato signals. Scale bar: 250 µm. (B) The uncropped brain section images for **Figure 8C**. Scale bar: 50 µm. (C) Immunohistochemistry with CC1 (an early OL marker) revealed that targeting dCas9-KRAB to the *Myrf* promoter (Pro, by G5 in **Figure 5A**), EC1 (by 5 in **Figure 5B**), EC2 (by 4 in **Figure 5B**), or EC1&2 (by 5 and 4 in **Figure 5B**, respectively)

downregulates CC1 signals of OL lineage cells in the mouse brain. Scale bar, 20 µm. **p* value $< 2.9 \times 10^{-19}$ by the cumulative binomial distribution function corrected by the Bonferroni procedure. (D) The uncropped brain section images for panel C. Scale bar: 50 µm.
